# Study of Extracts of *A. rusticana* as Corrosion Inhibitors of Mild Steel in 1 mol L^–1^ HCl Solution Using MALDI-TOF-MS

**DOI:** 10.1021/acsomega.5c07723

**Published:** 2025-10-31

**Authors:** Iendel Rubio do Nascimento, Klícia Carla de Santana de Lima, Lidilhone Hamerski, Eliane D’Elia

**Affiliations:** † Instituto de Química, 28125 Universidade Federal do Rio de Janeiro, Avenida Athos da Silveira Ramos, 149, Rio de Janeiro 21941-909, Brazil; ‡ Campus Duque de Caxias, Universidade Federal do Rio de Janeiro, Rodovia Washington Luiz, 19.593, km 104,5, Duque de Caxias 25240-005, Brazil; § Instituto de Pesquisas de Produtos Naturais, Universidade Federal do Rio de Janeiro, Carlos Chagas Filho—IPPN, 373, Rio de Janeiro 21941-853, Brazil

## Abstract

Corrosion studies were performed using extracts of *Armoracia rusticana* and their fractions through mass loss measurements, electrochemical impedance, and potentiodynamic polarization at concentrations of 100, 200, 400, 800, 1600, and 3200 mg L^–1^ in 1 mol L^–1^ HCl. The Fourier transform infrared, ESI-MS(±), MALDI-FT-ICR(±), and MALDI-TOF(±) techniques were used for the chemical characterization of the extracts and their fractions. The ethyl acetate fraction of the crude ethanolic extract (WEP-EtOAc) showed the highest inhibition efficiency, above 96%, verified by mass loss measurement with and without temperature variation and electrochemical tests. Scanning electron microscopy and X-ray photoelectron spectroscopy analyses were performed for the surface analysis. MALDI-TOF was also used for the ethyl acetate fraction of WEP, where the laser was applied directly to the metal plate under study. Even after washing, the glucosinolate chemical group was identified as the major component on the metal surface. This study suggests that glucosinolates are the possible main corrosion-inhibiting phytochemicals in the extract.

## Graphical abstract

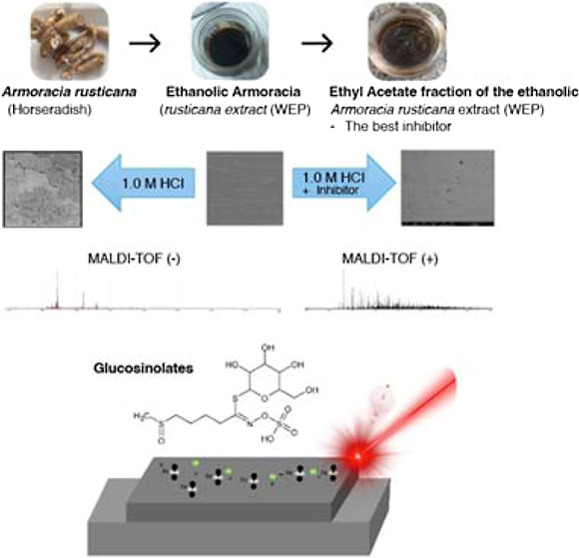

## 1 Introduction

Mild steel (MS) is one of the most used materials in industrial plants due to its low cost, excellent mechanical properties, and recyclability. However, this material does not exhibit chemical resistance and corrodes in acidic media such as those used in pickling, industrial cleaning, descaling, oil recovery, and petrochemical processes.^1,2^ HCl solutions have been widely used in corrosion studies by several authors, as they are aggressive and can be used to predict metal corrosion. Organic inhibitors have been introduced to mitigate the effects of corrosion. They are adsorbed on the metal surface through electrostatic attraction between the charged molecules and charged metal and/or by interacting pairs of uncharged electrons present in the inhibitory molecule with the metal. The stability of the formation of an inhibitor film on the surface will depend on the physicochemical characteristics of the inhibitory molecule, such as functional groups, aromaticity, steric effect, electronic density, and the charge of the metal surface.^3^

Using plant extracts as corrosion inhibitors is becoming an environmentally friendly, economically viable, and readily accessible alternative.^4^ Numerous studies have demonstrated the significant inhibitory action of various plant extracts in acidic media, achieving high inhibition percentages. Examples include *Carica papaya*, *Poinciana pulcherrima*, *Cassia occidentalis*, *Datura stramonium* seeds, and *Calotropis procera*, all of which have been reported to have inhibition percentages above 94%.^5^ Consequently, the search for new “green” or environmentally gentle corrosion inhibitors to replace synthetic options is actively pursued globally.^6^

Based on the above, our research investigated the potential of the crude extract and fractions of *Armoracia rusticana* to inhibit the corrosion of MS in a hydrochloric acid solution. *A. rusticana* belongs to the Brassicaceae family, originating from southeastern Europe and western Asia. It is widely cultivated in Eastern Europe and was introduced to Southern Brazil around 200 years ago by Central and Eastern European immigrants.^7^ The biological activities attributed to this species are due to the presence of numerous secondary metabolites, especially glucosinolates, in addition to isothiocyanates, organosulfur, flavonoid glycosides, organic/phenolic acids,^8–10^ coumarins, terpenes, vitamins, and amino acids.^11–13^ Glucosinolates comprise a β-D-thioglucoside group and an *N*-hydroxyiminosulfate ester with a variable side chain.^14^ Based on the precursor amino acid, they can be classified as aliphatic, indolic, or aromatic,^14^ are water-soluble,^15^ are relatively stable, but can undergo hydrolysis to isothiocyanate by the release of a β-thioglucosidase enzyme called myrosinase^16^ or in the intestine by plant and bacterial myrosinases.^17^ Figure 1 shows the general chemical structure of glucosinolate.

**Figure 1:**
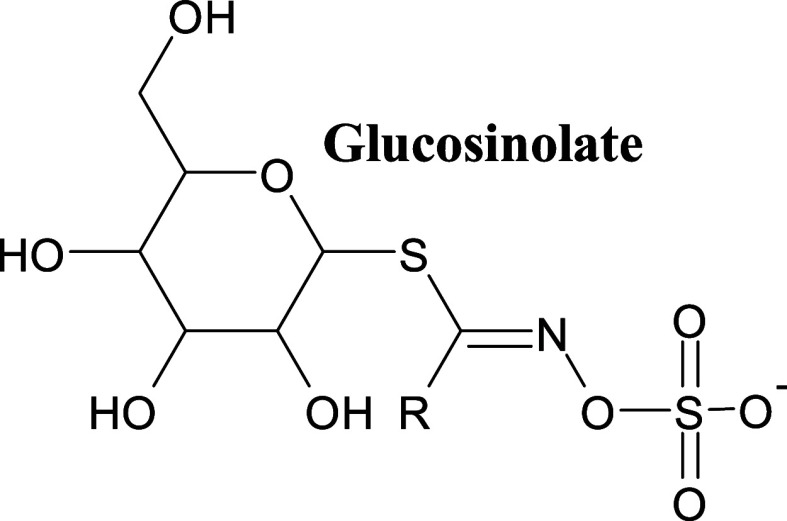
Representative chemical structure of glucosinolates.

Therefore, this research aims to investigate the efficacy of aqueous and ethanolic extracts of *A. rusticana* and their subsequent fractions as corrosion inhibitors for MS in a hydrochloric acid solution. This contributes to the growing body of knowledge about sustainable corrosion protection strategies.

## 2 Materials and Methods

### 2.1 Obtaining Crude Extracts of *A. rusticana* and Their Fractions

The plant root (*A. rusticana*) was received by mail after approximately 10 days of harvest in the Blumenau, Santa Catarina, and Southern Brazil region. It was placed in an oven at 40 °C and kept for 10 days to dry and ground to obtain a powder. The aqueous extract was prepared by decoction (3 days, with the addition of 500 mL of distilled water every 24 h), and the ethanolic extract was prepared by extraction (3 days, with the addition of 500 mL of anhydrous alcohol every 24 h). The crude aqueous extract (WAP) was obtained by lyophilization, and the crude ethanolic extract (WEP) was concentrated by using a rotary evaporator. The extracts were suspended in ethanol–water (6:4), partitions were performed with hexane (HEX) and ethyl acetate (EtOAc) (3 days—50 mL of solvent every 24 h), and the fractions, including the hydroethanolic fraction (residual-RES), were concentrated in a rotary evaporator and dried in a hood for 7 days.

### 2.2 Preparation of MS Specimens

The preparation of the working electrodes was carried out with MS plates with a mass percentage composition of C: 0.18; P: 0.04; S: 0.05; Mn: 0.30; Si: trace, and Fe: balance, with an approximate area of around 13 cm^2^ for mass loss and electrochemical tests, scanning electron microscopy (SEM) and matrix-assisted laser desorption/ionization–time of flight (MALDI-TOF), and approximately 6 cm^2^ for XPS (X-ray excited photoelectron spectroscopy) analyses. These plates were subjected to a treatment with wet sandpaper, using an AROPOL VV polisher (AROTEC), on damp sandpaper sheets (Jet 401 Norton and Carborundum) with grain sizes of 100, 320, 600 mesh for the mass loss, SEM, MALDI-FT-ICR, and XPS tests, and 100, 320, 600, and 1200 mesh for the electrochemical tests. All the MS specimens were washed with bidistilled water, degreased with ethanol, and dried in hot air. In the electrochemical tests, the surface area of the working electrode in contact with the corrosive medium was approximately 0.918 cm^2^.

### 2.3 Obtaining the Uninhibited and Inhibited Solutions

The uninhibited solution was an acidic solution of 1.0 mol L^–1^ HCl prepared by diluting analytical grade HCl (37 wt %) (Merck Co., Darmstadt, Germany) in double-distilled water. The experiments were performed under nonstirred and naturally aerated conditions. 100 mL of each extract and fraction solution was prepared in 1 mol L^–1^ HCl at different inhibitor concentrations: 100, 200, 400, 800, 1600, and 3200 mg L^–1^.

### 2.4 Infrared Spectroscopy

For each sample (WEP, WEP-HEX, WEP-EtOAc, WEP-RES, WAP, WAP-HEX, WAP-EtOAc, and WAP-RES), 10 mg was weighed. The spectra were obtained by a Nicolet 6700 Fourier transform infrared (FT-IR) spectrometer with a DTGS KBr detector and a KBr beam splitter, measured in the 4000 to 400 cm^–1^ range.

### 2.5 Mass Spectrometry—MS-ESI-FT-ICR(±) and MS-MALDI-TOF-FTICR(±)

Hybrid Quadrupole-Orbitrap MS—Thermo Qexactive (Thermo Scientific) with electrospray ionization source, ESI–MS, direct infusion with 10, resolution 140,000 (fwhm) for *m*/*z* 400, mode (±), spray voltage: 3.6­(+) and 3.3(−) Kv, S-Lens voltage: 60 V, capillary temperature: 280 °C, sheath gas: 10 and auxiliary gas: 0. ESI-QTOF Maxis Impact (Bruker): 50 *m*/*z* low mass quadrupole. External calibration with 100 μM sodium formate in water/acetonitrile 1:1, scan range: 20–1200 *m*/*z*, acquisition rate: 1 Hz, Nebulizer pressure: 0.4 bar, dry gas: 4.0 L min^–1^, dry temperature: 180 °C, capillary voltage: 2000 V, end plate offset: 500 V, the WAP sample was solubilized in 500 μL of water, and the WEP sample was solubilized in 500 μL of ethanol and then diluted 1000× in methanol. Autoflex Speed (MALDI—TOF, Bruker), polarity: positive, mass range: 80 to 2000 *m*/*z*, suppression up to 100 Da, TOF acquisition mode: reflector, calibration with red phosphorus cluster masses. Samples were diluted 50× in 0.1% trifluoroacetic acid in water: acetonitrile 1:1 and then mixed 1:1 with a 10 mg mL^–1^ solution of 2,5-dihydroxybenzoic acid in 0.1% trifluoroacetic acid in water: acetonitrile 1:1, then applied to the MALDI plate (1 μL). Solarix XR (FT-ICR, Bruker)—polarity: positive and negative, mass range: 75 to 1000 *m*/*z*, time domain data set size: 2 MW, resolution 99,000 for *m*/*z* 400. The sample was dried in a roto evaporator and resuspended in 50 μL of water, then mixed 1:1 with a 10 mg mL^–1^ solution of 2,5-dihydroxybenzoic acid in 0.1% trifluoroacetic acid in water: acetonitrile 1:1, and then applied to the MALDI plate (1 μL). To prepare the metal plates, 2 μL of the matrix was applied to the “washed” and “unwashed” metal plates. The matrix used 10 mg mL^–1^ 2,5-dihydroxybenzoic acid solution in 0.1% trifluoroacetic acid in water: acetonitrile 1:1. Plates without applying a matrix were also analyzed.

### 2.6 Scanning Electron Microscopy

Scanning electron microscopy was performed by Bruker Nano GmbH Berlin, Germany, Esprit 2.x, pulse density: 38,298, primary energy: 20, beam current: −1, take off angle: 35, tilt angle: 0, azimuth angle: 45, detector type: XFlash 660, window type: slew AP3.3, detector thickness: 0.45, Si dead layer: 0.029, Mn fwhm: 145.5, Fano factor: 0.133, calibration, lin.: 4.999, calibration, abs.: −477.754, channels: 4096. The analyses were performed at CETEM (Center for Mineral Technology).

### 2.7 X-ray Excited Photoelectron Spectroscopy

XPS analyses were performed on a ThermoScientific ESCALAB 250Xi Spectrometer equipped with a hemispherical energy analyzer and operated in an ultrahigh vacuum. XPS spectra were collected by using an Al X-ray source (Kα = 1486.7 eV). Full-scan spectra were recorded with a pass energy of 100 eV and an energy step of 1 eV. High-resolution spectra were obtained with a pass energy of 25 eV and an energy step of 0.1 eV. Energy scale calibration was performed using the Au 4f line (84.0 eV). Data acquisition and processing were performed using Thermo Avantage Software. The sensitivity factors for each element and energy level were considered to quantify the full-scan spectrum. A Lorentzian/Gaussian ratio of 30:70 was applied to adjust the peaks of the high-resolution spectra.

### 2.8 Gravimetric Measurements

The mass loss measurements were obtained according to ASTM G31-72 ^18^ by immersing the specimens in the corrosive medium. The masses of the specimens were determined using an analytical balance with an accuracy of 0.1 mg before and after immersion, and their area was measured using a digital calliper before immersion. After immersion, the specimens were washed with distilled water, degreased with ethanol, and dried in a hot air oven. Three specimens were used for each immersion condition. The effect of time and extract concentration on the corrosion rate of MS in 1.0 mol L^–1^ HCl was observed for 2, 8, and 24 h at room temperature in the absence and presence of 100, 200, 400, 800, 1600, and 3200 mg L^–1^ of the extracts and the presence of 400 mg L^–1^ of their fractions. Equations 1 and 2 were used to calculate the corrosion rate expressed in g cm^–2^ h^–1^ and the inhibition efficiency (IE %), respectively.

Mass loss is the most reliable and accurate method for determining corrosion rate and inhibitor inhibition efficiency.^19^

1 $$W_{corr}=\frac{\Delta m}{t\cdot A}$$

wherein Δ*m* represents mass loss (given in mg), *A* represents the surface area of the specimen (in cm^2^), and *t* represents the immersion time (in hours).

2 $${IE}_{WL}\;\%=\frac{W_{corr,0}-W_{corr}}{W_{corr,0}}\times 100$$

*W*_corr,0_ and *W*_corr_ represent the corrosion rates of the MS coupons in the absence and presence of inhibitors, respectively. The corrosion rate (*W*_corr_) is shown in mg cm^–2^ h^–1^.

### 2.9 Electrochemical Procedure

All electrochemical measurements were performed in a three-electrode Pyrex glass cell: MS as a working electrode, a saturated calomel electrode as a reference, and a large surface area platinum screen as a counter electrode inside a Faraday cage. The tests were performed in an AUTOLAB-PGSTAT 128 N potentiostat/galvanostat with a Metrohm impedance module coupled to a computer. The software used was Nova 2.1.6 (Metrohm). All experiments were performed in 100 mL of the corrosive medium without stirring and under naturally aerated conditions maintained at room temperature in the absence and presence of the crude extracts and their fractions in triplicate. The electrolyte was a 1.0 mol L^–1^ HCl solution. Before each experiment, the electrode was freely corroded and its open circuit potential (OCP) was recorded as a function of time for 5000 s. After this time, a steady-state OCP value was obtained. Electrochemical impedance measurements were obtained on the stabilized OCP with the following parameters during data acquisition: frequency range of 100 kHz to 10 mHz, with 10 points per decade, and perturbation amplitude of 10 mV in triplicate. After the electrochemical impedance measurements, cathodic and anodic polarization curves were obtained by varying the potential from −300 to +300 mV to the OCP with a scan rate of 1 mV s^–1^.

## 3 Results and Discussion

### 3.1 Obtaining WEP, WAP Extracts, and Their Respective Hexane, Ethyl Acetate, and Residual Fractions

Table 1 shows the mass values obtained in the ethanolic and aqueous extractions of the *A. rusticana* plant and its fractions obtained from partition with hexane and ethyl acetate.

**Table 1:** Mass Values Obtained from the Ethanolic and Aqueous Extractions of the *A. rusticana* Plant and its Fractions Obtained from the Partition with Hexane and Ethyl Acetate

sample	sample quantity	dry material 40 °C 10 days	crude extract	hexane fraction	ethyl acetate fraction	residual fraction
A. rusticana	350 g	188.3791 g	WEP: 29.4231 g	0.8108 g	1.5608 g	9.6049 g
A. rusticana	500 g	379.2132 g	WAP: 38.4325 g	0.0434 g	3.3903 g	9.2858 g

### 3.2 Characterization of the Ethanolic Extract of *A. rusticana* (WEP) and its Fractions

#### 3.2.1 FT-IR, ESI-FT-ICR, ESI-Q-TOF, and MALDI-TOF Modes (±)

The FT-IR spectra of the ethanolic extract of *A. rusticana* (WEP) and its corresponding fractions (hexane, ethyl acetate, and residual) were acquired to identify characteristic functional groups. The spectra are presented in Figure 2. The WAP and its fractions (hexane, ethyl acetate, and residual) FTIR spectra are shown in Figure S1 of the Supporting Information.

**Figure 2:**
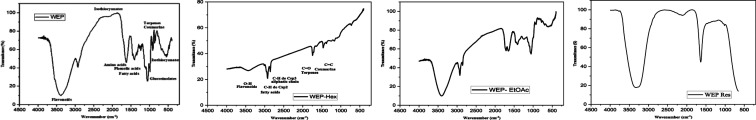
FTIR spectrum of the extract of *A. rusticana* in ethanol (WEP) and its hexane, ethyl acetate, and residual fractions.

The FT-IR spectrum of the crude ethanolic extract (WEP) exhibited a profile consistent with the presence of glucosinolates, as previously reported in the literature.^20^ A broad absorption band centered at approximately 3400 cm^–1^ was observed, indicative of O–H stretching vibrations, suggesting the presence of hydroxyl-containing compounds such as phenolic compounds and carbohydrates. Aliphatic C–H stretching vibrations were observed at 2932 cm^–1^. Characteristic C–O stretching vibrations, associated with glycosidic linkages in carbohydrates, were detected at 1255 cm^–1^ and 991 cm^–1^. A prominent band at 1630 cm^–1^ was assigned to the C=N-sulfate stretching vibration, a key feature of glucosinolates.^22^

The FT-IR spectra of the fractionated extracts revealed distinct functional group compositions. A broadband of around 3428 cm^–1^ was observed in all fractions, confirming the presence of the O–H stretching vibrations. The hexane fraction peaked at 1743 cm^–1^, attributed to C=O stretching vibrations characteristic of esters and aldehydes.^23^ The ethyl acetate fraction showed a peak at 1712 cm^–1^, indicative of ketone carbonyl stretching, and a peak at 1655 cm^–1^, which could be attributed to either conjugated carbonyl stretching or C=N (imine) stretching with a conjugation effect similar to C=O.^24^ Further peaks within the ethyl acetate fraction included a signal at 1056 cm^–1^, potentially corresponding to S=O stretching in sulfoxides or O–H stretching in alcohols, a signal at 1412 cm^–1^ assigned to S=O stretching in sulfates, and a signal at 950 cm^–1^ related to S–O stretching.^25^

The residual fraction displayed a weak band at 1636 cm^–1^, potentially indicative of an N-substituted amide group. Absorption bands characteristic of glucosinolates and isothiocyanates were observed at 2114 cm^–1^ (asymmetric N=C=S stretching) and 1010 cm^–1^ (symmetric N–C–S stretching).^26–28^ The band at 2114 cm^–1^ is consistent with compounds such as sulforaphane^29^ and cheirolin.^30^

FTIR spectra of WAP and fractions are presented in the Supporting Information (Figure S1A–D). Mass spectra were obtained using ESI(±) and MALDI(±) techniques, with a quadrupole analyzer and a TOF analyzer, for the ethanolic and aqueous extracts of *A. rusticana* as well as its hexane, ethyl acetate, and residual fractions. The signals identified in the spectra and possible chemical structures are presented in the Supporting Information (Figures S2–S9 and Tables S1–S4), confirming the chemical groups identified by FTIR.^22^

### 3.3 Mass Loss Measurements

Table 2 shows the corrosion rate and the inhibition efficiency for MS in 1 mol L^–1^ HCl with and without the presence of inhibitor at concentrations of 100, 200, 400, 800, and 1600 mg L^–1^ for WEP and 100, 200, 400, 800, 1600, and 3200 mg L^–1^ for WAP, in the periods of 2, 8, and 24 h of immersion and at the concentration of 400 mg L^–1^ for the ethyl acetate and residual fractions of WEP (WEP-EtOAc, WEP-RES) and WAP (WAP-EtOAc, WAP-RES) in 2 h at room temperature. The crude extracts showed an increased inhibition efficiency as the concentration and immersion time increased. All extracts showed an inhibition efficiency above 90% in 2 h of immersion; WEP presented an IE of 91.0% at 800 mg L^–1^ and WAP 91.6% at 3200 mg L^–1^. During the 8 h immersion period, we observed an increase in the inhibition efficiency of all inhibitors at all concentrations studied due to the decrease in the corrosion rate caused by the more significant adsorption of molecules present in the WEP and WAP extracts, with a maximum IE of 94.0 and 92.9%, respectively. After 24 h of immersion, WEP presented a greater inhibition efficiency, reaching 94.7%. The ethanolic extract of *A. rusticana* WEP was more efficient than its aqueous extract. This shows that the molecules responsible for the most significant inhibitory effect are more concentrated in the ethanolic phase of *A. rusticana* than in the aqueous extract. The inhibitors delay the dissolution of MS in 1 mol L^–1^ HCl solution at all concentrations used, and this effect increases as the inhibitor concentration increases.^31^ The ethyl acetate and residual fractions were studied at a concentration of 400 mg L^–1^. The ethyl acetate fraction of WEP exhibited the highest inhibition efficiency, reaching 97.5% and 98.1% after 2 and 24 h of immersion, respectively, thus demonstrating strong anticorrosive performance even over prolonged exposure. The residual fraction of WEP also showed significant activity, with an IE of 87.3%. These results confirm that the substances responsible for the inhibitory effect of the crude extract of WEP were successfully extracted and concentrated in the ethyl acetate fraction. Similarly, the ethyl acetate fraction of WAP showed a substantial 80.1% IE, while the residual fraction showed 76.3%. Figure 3 shows the relationship between the corrosion rate and the concentrations of the inhibitors for different immersion times. The results show that the inhibition efficiency of the extracts increases with time and remains high for up to 24 h in a highly corrosive medium (1 mol L^–1^ HCl). This demonstrates that the phytochemicals responsible for the inhibitory effect are stable.^32^ From 800 mg L^–1^, the IE remains relatively constant after 8 h. Additionally, these compounds should feature a high electron density due to aromatic rings and nonbonding electrons from heteroatoms (O, N, and S, in our case) and protonated species in their structure.^33^ These functional groups adsorb onto the metal surface. This process effectively blocks the more active corrosion sites, preventing contact between the metal and the corrosive medium.^34^ As a result, the extracts remained stable and adsorbed on the MS surface during this time.

**Table 2:** Corrosion Rates Obtained from the Mass Loss of MS in 1 mol L^–1^ HCl, Containing Various Concentrations 100, 200, 400, 800, 1600, and 3200 mg L^–1^ of Ethanolic and Aqueous Extract of *A. rusticana* and its Ethyl Acetate and Residual Fractions, in the Periods of 2, 8, and 24 h

sample	[inhibitor] (mg L^–1^)	2 h		8 h		24 h	
		*W*_corr_ (mg cm^–2^ h^–1^)	IE ± SD_IE_ (%)	*W*_corr_ (mg cm^–2^ h^–1^)	IE ± SD_IE_ (%)	*W*_corr_ (mg cm^–2^ h^–1^)	IE ± SD_IE_ (%)
blank	**0**	1.2688 × 10^–3^		1.4396 × 10^–3^		1.7824 × 10^–3^	
WEP	**100**	3.5016 × 10^–4^	72.4% ± 2.0	3.4085 × 10^–4^	76.3% ± 2.0	3.4574 × 10^–4^	80.6% ± 2.6
WEP	**200**	2.3807 × 10^–4^	81.2% ± 2.2	2.3727 × 10^–4^	83.5% ± 2.4	3.0208 × 10^–4^	83.1% ± 1.3
WEP	**400**	1.9024 × 10^–4^	85.0% ± 1.1	2.0980 × 10^–4^	85.4% ± 0.5	2.2644 × 10^–4^	87.3% ± 2.3
WEP	**800**	1.1422 × 10^–4^	91.0% ± 1.6	1.1625 × 10^–4^	91.9% ± 0.3	1.1532 × 10^–4^	93.5% ± 1.6
WEP	**1600**	8.9017 × 10^–5^	93.0% ± 1.1	8.6346 × 10^–5^	94.0% ± 1.4	9.4759 × 10^–5^	94.7% ± 0.5
blank	**0**	8.8139 × 10^–3^					
WEP-EtOAc	**400**	2.1652 × 10^–4^	97.5% ± 1.0				98.1% ± 0.2
WEP-RES	**400**	1.1194 × 10^–3^	87.3% ± 1.5				
blank	**0**	1.4984 × 10^–3^		2.5593 × 10^–3^		2.8421 × 10^–3^	
WAP	**100**	6.1534 × 10^–4^	58.9% ± 2.3	1.0196 × 10^–4^	60.2% ± 1.4	9.7085 × 10^–4^	65.8% ± 1.5
WAP	**200**	5.7429 × 10^–4^	61.7% ± 1.7	9.6271 × 10^–4^	62.4% ± 1.2	9.6423 × 10^–4^	66.1% ± 2.5
WAP	**400**	5.3976 × 10^–4^	64.0% ± 0.7	8.6766 × 10^–4^	66.1% ± 1.7	6.8602 × 10^–4^	75.9% ± 2.4
WAP	**800**	2.5917 × 10^–4^	82.7% ± 0.5	3.9546 × 10^–4^	84.5% ± 1.9	4.3706 × 10^–4^	84.6% ± 0.3
WAP	**1600**	2.1422 × 10^–4^	85.7% ± 1.3	3.3822 × 10^–4^	86.8% ± 1.3	3.0782 × 10^–4^	89.2% ± 0.9
WAP	**3200**	1.2588 × 10^–4^	91.6% ± 1.2	1.8209 × 10^–4^	92.9% ± 1.3	1.8593 × 10^–4^	93.5% ± 0.9
blank	**0**	8.8139 × 10^–3^					
WAP-EtOAc	**400**	1.7569 × 10^–3^	80.1% ± 1.8				
WAP-RES	**400**	2.0910 × 10^–3^	76.3% ± 0.3				

**Figure 3:**
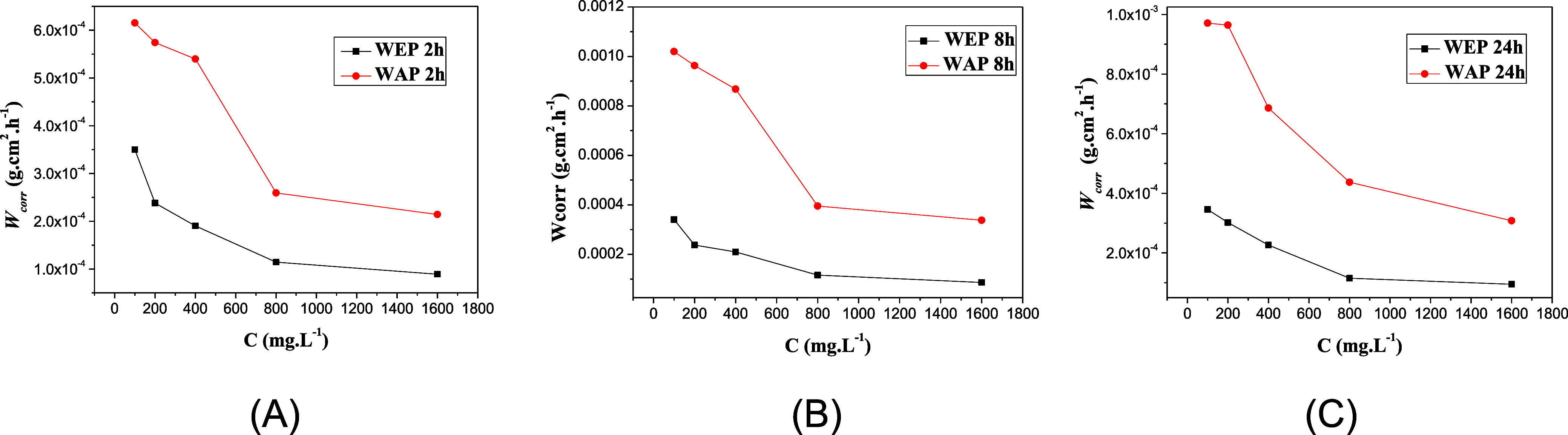
Corrosion rate of MS in 1 mol L^–1^ HCl solution in the absence and presence of WEP and WAP for different concentrations (100, 200, 400, 800, and 1600 mg L^–1^) after 2 h (A), 8 h (B), and 24 h (C) of immersion at 25 °C.

The influence of the temperature on the inhibitory action of the extracts was studied using 200 mg L^–1^ of the inhibitors WEP and WAP for 2 h of immersion time. The IE results are shown in Table 3, and the activation energy values were calculated using eq 3 by plotting the Arrhenius graphs (Figure 4).

3 $$\log W_{corr}=\log A-\frac{Ea}{2.303RT}$$

where *W*_corr_ is the corrosion rate (in mg cm^–2^ h^–1^), *A* is the Arrhenius constant, *Ea* is the activation energy, *R* is the gas constant (in J K^–1^ mol^–1^), and *T* is the absolute temperature (in K).

**Table 3:** Corrosion Rate Values and Inhibition Efficiency of Inhibitors at Different Temperatures for 2 h of Immersion Time and 200 mg L^–1^ of Inhibitor

sample	25 °C		35 °C		45 °C		55 °C	
	*W*_corr_ (mg cm^–2^ h^–1^)	IE ± SD_IE_ (%)	*W*_corr_ (mg cm^–2^ h^–1^)	IE ± SD_IE_ (%)	*W*_corr_ (mg cm^–2^ h^–1^)	IE ± SD_IE_ (%)	*W*_corr_ (mg cm^–2^ h^–1^)	IE ± SD_IE_ (%)
blank	1.7504		3.7092		5.1085		10.1253	
WEP 200 mg L^–1^	0.3818	78.2% ± 1.0	0.6941	81.3% ± 1.3	0.9369	81.7% ± 0.3	2.0586	79.7% ± 2.2
blank	1.7504		3.7092		5.1085		10.1253	
WAP 200 mg L^–1^	0.6901	61.5% ± 1.3	0.9502	64.9% ± 1.3	1.8153	65.1% ± 0.2	3.5462	58.9% ± 0.4

**Figure 4:**
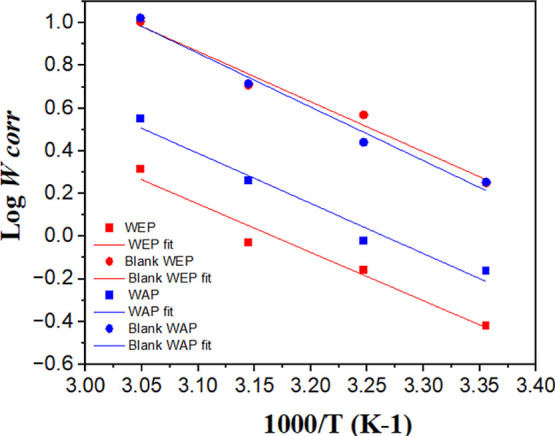
Arrhenius graphs for MS corrosion in 1 mol L^–1^ HCl solution in the absence and presence of 200 mg L^–1^ of WEP and WAP at 25, 35, 45, and 55 °C after 2 h of immersion.

The corrosion rate rises with increasing temperature in both the presence and absence of inhibitors. However, this increase is more pronounced in uninhibited acid solutions, suggesting chemical adsorption. The maximum IE for WEP and WAP extracts occurred at 45 °C with 81.7% and 65.1%, respectively. This increase in efficiency is a consequence of the adsorption of more inhibitor molecules on the metal surface, forming a still more protective film.^35^ At 55 °C, IE decreases slightly for WEP and WAP, reaching 79.7% and 58.9%, respectively (Table 3 and Figure 4). The increase in temperature can lead to a rise in the desorption rate of the inhibitor molecules and/or even the decomposition of part of these molecules. In the present case, the degradation of the inhibitory molecule is more likely since the interaction should be chemical between the inhibitory molecules and the MS surface.^36–38^

The presence of the inhibitor caused a decrease in the activation energy (Table 4), characteristic of chemisorption, which presents forces comparable to those of chemical bonds. The *Ea* values obtained corroborate that the metal-inhibitor adsorption process is defined as chemisorption.

**Table 4:** Values Referring to the Arrhenius Graph in Figure 3

*Ea* (kJ mol^–1^)	line equation	*R* ^2^	sample
45.5	*y* = −2.3499*x* + 8.1505	*R* ^2^ = 0.9807	blank
43.4	*y* = −2.2684*x* + 7.1836	*R* ^2^ = 0.9681	WEP
48.2	*y* = −2.5181*x* + 8.6637	*R* ^2^ = 0.9820	blank
45.0	*y* = −2.3482*x* + 7.6682	*R* ^2^ = 0.9690	WAP

During the inhibitory process, the inhibitory molecules adsorb on the metal surface over time, while the corrosion rate decreases. The adsorption of an organic adsorbate between the metal/solution interface can be considered as a replacement adsorption process between the inhibitory molecules in the aqueous solution, Inh­(soln), and the water molecules on the metal surface, H_2_O_(ads)_ ^39^

$${Inh}_{\left(sol\right)}+{xH}_{2}O_{\left(ads\right)}↔{Inh}_{\left(ads\right)}+{xH}_{2}O_{\left(sol\right)}$$

Inh_(ads)_ are the inhibitory molecules adsorbed on the metal surface, H_2_O_(soln)_ is the water molecules in the aqueous solution, and *x* represents the number of water molecules displaced by an inhibitory adsorbate molecule on the metal surface. The interaction between the inhibitory molecules and the substrate was studied by using adsorption isotherm models.

From the inhibition efficiency data (eq 4), it was possible to determine the degree of coverage of the metal surface (θ) as a function of the inhibitor concentration. The experimental results were adjusted to different adsorption isotherms, Langmuir (eq 5), Temkin (eq 6), Flory–Huggins (eq 7), and El-Awady (eq 8)­

4 $$\theta =\frac{IE}{100}$$

5 $$\frac{C}{\theta }=\frac{1}{K}+C$$

6 $$\theta =\left(\frac{-2.303}{2a}\right)\log K+\left(\frac{-2.303}{2a}\right)\log C$$

7 $$\log\left(\frac{\theta }{C}\right)=\log K+x\;\log\left(1-\theta \right)$$

8 $$\log\left(\frac{\theta }{\left(1-\theta \right)}\right)=\log K+y\;\log C$$

where *C* is the inhibitor concentration, *K* is the adsorption constant, *a* is the lateral parameter of interaction between adsorbed molecules, *x* is the number of adsorbed water molecules substituted by inhibitor molecules, and *y* is the number of adsorbed inhibitory molecules in an active site.^40^

Langmuir’s theory is founded on several key assumptions about adsorption. It posits that adsorption forms a monolayer on the surface, occurs at specific, homogeneous sites on the adsorbent, and that the energy of adsorption remains constant. This energy does not depend on the occupancy of the active sites on the adsorbent. Furthermore, all adsorption sites are considered identical and energetically equivalent, with no interaction between the adsorbed molecules and neighboring sites.^41^

The Temkin isotherm model proposes that the heat of adsorption decreases linearly for all the molecules covering the adsorbent, depending on the interactions between the adsorbed molecules. A positive value (*a* > 0) indicates attraction, while a negative value (*a* < 0) signifies repulsion. On the other hand, the Flory–Huggins isotherm suggests that the number of water molecules displaced by inhibitor molecules, represented by “*x*”, could differ from one. If *x* is more significant than one, it indicates that an inhibitory molecule has substituted more than one molecule of H_2_O­(ads). Awady et al. further elaborate on how many inhibitory molecules can occupy one active site, as parameter *y* indicates. If *y* is less than one, a single inhibitory molecule was adsorbed onto multiple active sites. Thus, *y* represents the number of inhibitor molecules occupying an active site on the metal surface.^3,42,43^ The isotherms and equations are presented in Supporting Information in Figures S10 and S11 and Table S5.

The Langmuir isotherm model shows an *R* ^2^ value greater than 0.998 for all immersion times and WEP and WAP inhibitors. However, the angular coefficient exceeds 1, indicating a deviation from the model’s ideal conditions. These results imply an interaction between the adsorbed inhibitor molecules, or that the relationship between the active sites for each adsorbed molecule differs from unity. The Temkin isotherm provides insight into the molecular interactions within the adsorbed layer. The value of *a* was negative under all immersion time conditions (Table S5), suggesting that the dominant interaction in the layer is repulsive. The Flory–Huggins model is vital for assessing deviations from ideality in the Langmuir model. The *x* value was higher than unity (Table S5), showing that one inhibitor molecule replaced more than one water molecule. Additionally, the El-Alwady model aids in predicting the number of inhibitor molecules that occupy a single active site. The *y* value was lower than 1 for the extracts studied (Table S5), indicating that a single inhibitor molecule occupies more than one active site.

Adsorption isotherms are employed to calculate the adsorption constant, allowing us to determine the Gibbs free energy of adsorption. This value reflects the interaction between the metal and the inhibitor. Typically, values of Δ*G* ^°^_ads_ more positive than −20 kJ/mol indicate physical adsorption, while values more negative than −40 kJ/mol signify chemical adsorption. Values between these ranges are classified as mixed-type adsorption with physical and chemical interaction with the metal surface.^44,45^ The Langmuir isotherm model proved to be the most suitable in this study, producing a determination coefficient value close to 1. However, all the other isotherms demonstrated *R* ^2^ values above 0.9. The adsorption equilibrium constant offers insights into the strength of the adsorption force between the inhibitor and the MS surface. A higher *K* value signifies denser adsorption, leading to more effective inhibition.^46,47^ However, according to ref 44, using the Langmuir equation to calculate the Δ*G* ^°^_ads_ for solutions containing undefined inhibitors (as natural extracts) is impossible since the molecular mass of the inhibitory molecule is unknown.

Although the literature reports a high concentration of glucosinolates in *A. rusticana*,^21^ plant extracts present different classes of compounds, and many molecules in this mixture, with electronegative heteroatoms such as N, O, and S, which can be readily adsorbed on the metal surface, together with alternating double bonds and aromatic rings, creating adsorption centers and the following decreasing order of adsorption efficiency of heteroatoms is P > S > O > N.^48^

### 3.4 Electrochemical Measurements

Figure 5 illustrates the determination of the OCP for MS in the presence and absence of an inhibitor. The measurements were conducted over 5000 s, achieving stability around 4000 s. The OCP values recorded were −0.507 V, −0.491 V, −0.494 V, −0.488 V, −0.501 V, −0.496 V, −0.480 V, −0.501 V, and −0.504 V at various concentrations of WEP: 0, 100, 200, 400, 800, and 1600 mg L^–1^, respectively, as well as for 400 mg L^–1^ WEP-EtOAc, and 400 mg L^–1^ WEP-RES. For WAP, the potential values reached were −0.507, −0.486, −0.483, −0.486, −0.490, −0.494, −0.502, −0.490, and −0.487 V, respectively, at concentrations of 0, 100, 200, 400, 800, 1600, and 3200 mg L^–1^ WAP, 400 mg L^–1^ of WAP-EtOAc, and WAP-RES. The extracts and their fractions stabilized at more anodic potentials, with a maximum shift of +27 mV and +24 mV for WEP and WAP, respectively, presenting mixed inhibitor characteristics, suggesting the formation of a protective film on the metal surface, according to the study of *A. rusticana* in 0.5 mol L^–1^ sulfuric acid.^49^

**Figure 5:**
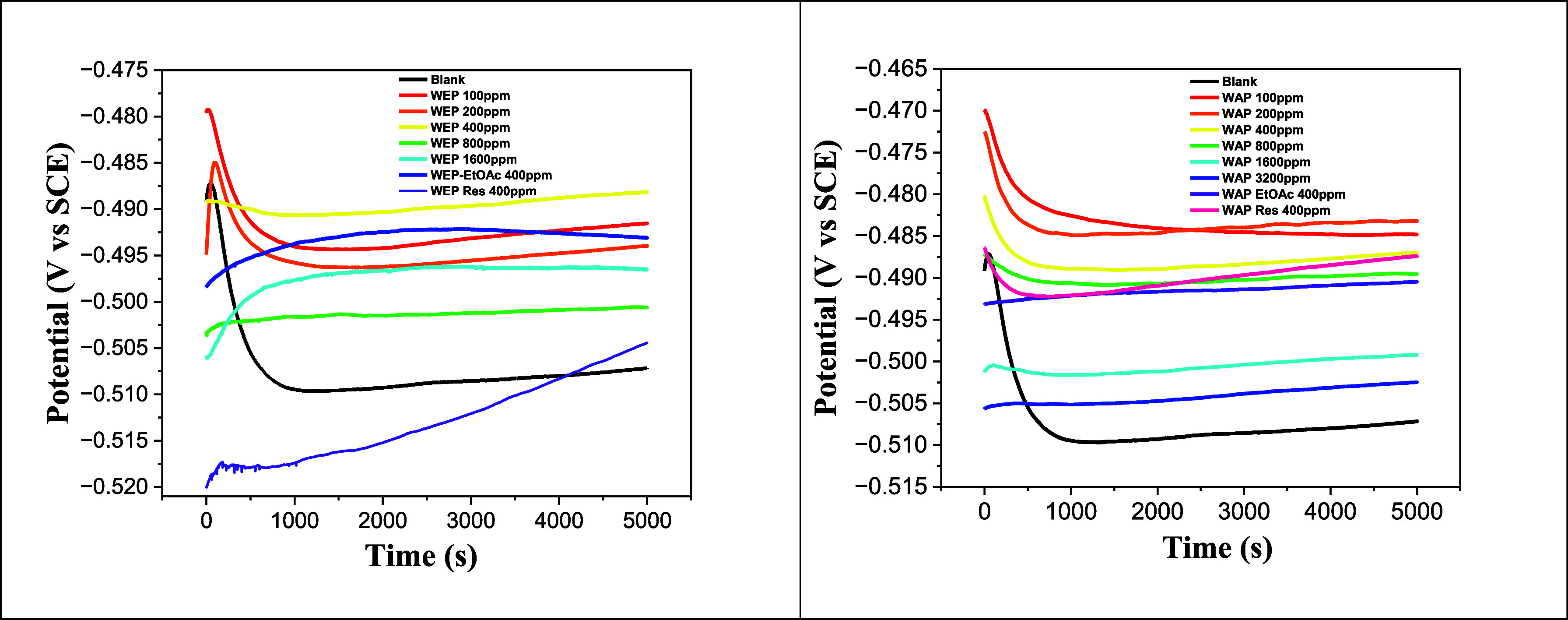
OCP versus immersion time for MS in 1.0 mol L^–1^ HCl with and without an inhibitor.

The blank curve shows a decrease in potential in the first 1000 s, mainly due to the dissolution of the oxide film, and then the corrosion of the MS caused by the acid. In the presence of the inhibitor, we observed the same curve shape but a more prolonged stabilization at more noble potentials, probably caused by the adsorption of phytochemicals in the solution.^40^

The Nyquist plot and the Bode plots for MS in 1.0 mol L^–1^ HCl solution with and without an inhibitor are illustrated in Figure 6. In the absence of an inhibitor (blank), the diagram shows only one capacitive loop related to the charge transfer resistance and the capacitance of the electrical double layer. With the addition of the inhibitor, this capacitive loop increases significantly, indicating that the inhibitor forms a protective film on the surface of MS, hindering the action of the H^+^ ions. The impedance modulus increases at low frequencies with higher inhibitor concentrations. This observation suggests that as the concentration of the inhibitor increases, more inhibitory molecules adsorb onto the surface of the MS, enhancing the degree of surface coverage. This expanded coverage hinders the charge transfer process between the MS and the solution. The maximum phase angle also shows a significant increase in the presence of the inhibitor, reaching about 70° with the addition of WEP (1600 mg L^–1^) and WAP (3200 mg L^–1^). This indicates that the enhanced capacitive behavior is a result of the formation of a protective film. Moreover, a small inductive loop appears with the addition of the high inhibitor concentrations, whose origin may stem from the relaxation of inhibitory species on the surface of MS or intermediary species involved in the corrosion reaction.^39,40,50^

**Figure 6:**
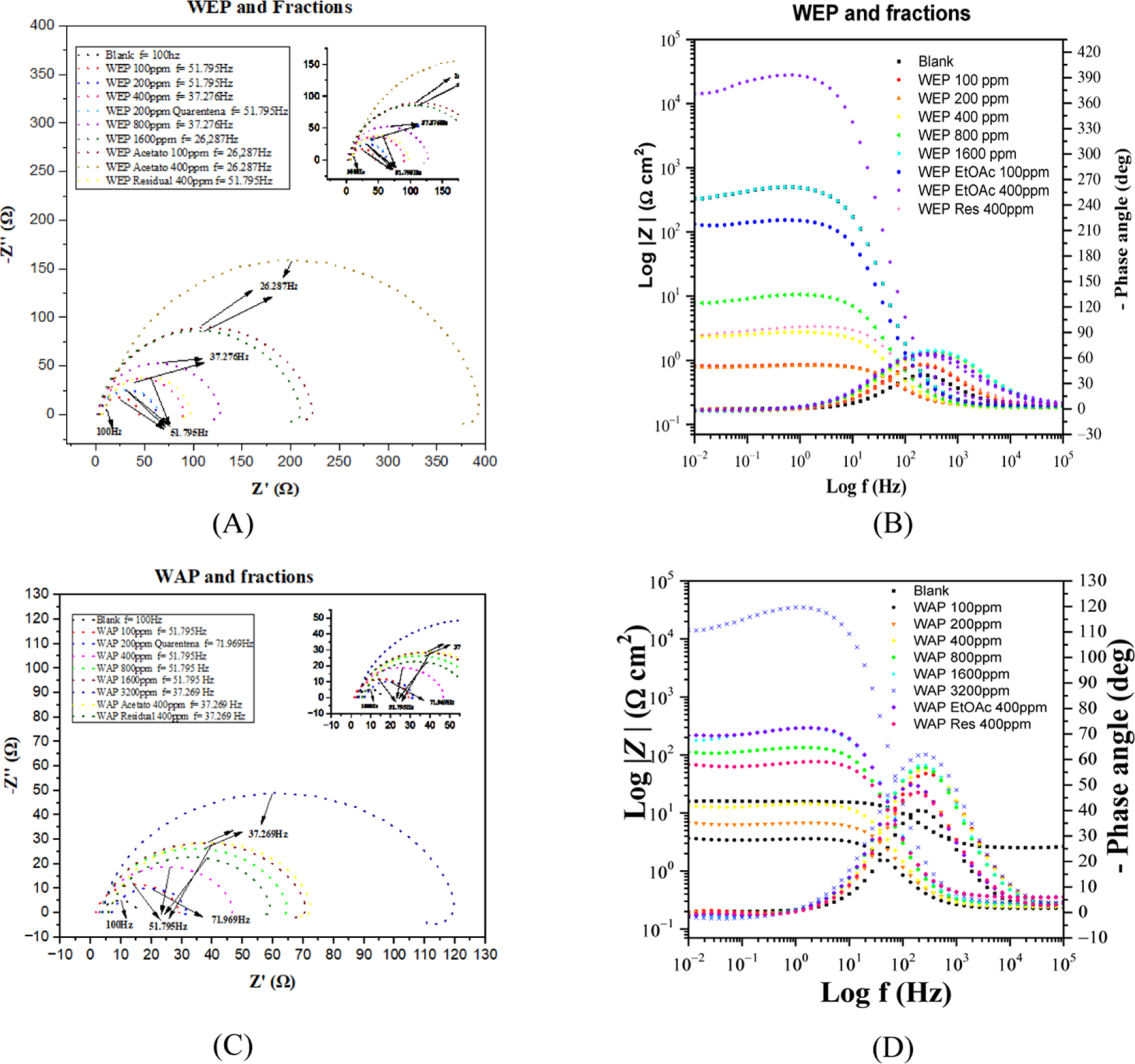
Nyquist and Bode plots of MS in 1 mol L^–1^ HCl in the absence and presence of WEP (A and B) and WAP (C and D) at different concentrations and their fractions.

Table 5 summarizes the electrochemical parameters obtained from the electrochemical impedance diagrams presented in Figure 6. The increase in the concentration of the inhibitor causes a decrease in the capacitance of the electrical double layer (*C*_dl_) and an increase in the charge transfer resistance (*R*_ct_). The *C*_dl_ values were obtained using eq 9. The decrease in *C*_dl_ may be caused by a reduction in the local dielectric constant or by an increase in the thickness of the electrical double layer. These results indicate that the added extracts altered the electric double-layer structure, implying that the inhibitory molecules interacted through adsorption at the metal/solution interface.

9 $$C_{dl}=\frac{1}{2\pi f_{\max}R_{ct}}$$

where *C*_dl_ is the electrical double-layer capacitance, *R*_ct_ is the charge transfer resistance, and *f*_max_ is the frequency at which the imaginary component of the impedance is maximal.

**Table 5:** Electrochemical Parameters for MS in 1 mol L^–1^ HCl in the Absence and Presence of WEP, WAP, and Their Fractions

sample	[inhibitor] (mg L^–1^)	*C*_dl_ (μF cm^–2^)	*R*_ct_ (Ω cm^–2^)	IE(%) ± SD_IE_ (%)	*f*_max_ (Hz)
blank	0	133	12.0		100
WEP	100	74.9	41.0	70.7 ± 2.0	51.8
WEP	200	54.0	56.9	78.9 ± 0.3	51.8
WEP	400	48.0	89.0	86.5 ± 1.2	37.3
WEP	800	33.8	126	90.5 ± 0.4	37.3
WEP	1600	29.2	208	94.2 ± 2.1	26.3
WEP-EtOAc	100	23.8	254	95.3 ± 0.4	26.3
WEP-EtOAc	400	14.7	404	97.0 ± 0.5	26.8
WEP-RES	400	33.8	91.0	86.8 ± 0.2	51.8
WAP	100	111.9	27.5	56.4 ± 1.0	51.8
WAP	200	97.4	31.6	62.0 ± 2.0	51.8
WAP	400	76.8	40.0	70.0 ± 1.2	51.8
WAP	800	50.1	61.3	80.4 ± 1.2	51.8
WAP	1600	46.1	66.7	82.0 ± 1.0	51.8
WAP	3200	36.9	116	89.7 ± 1.2	37.3
WAP-EtOAc	400	63.6	67.1	82.1 ± 0.2	37.3
WAP-RES	400	54.9	56.0	78.6 ± 0.7	51.8

The inhibition efficiency data derived from the *R*_ct_ values align with the results obtained from mass loss measurements (Tables 2 and 5). The maximum inhibition efficiencies recorded were 94.2% for 1600 mg L^–1^ WEP and 89.7% for 3200 mg L^–1^ of WAP. WEP-EtOAc at 100 mg L^–1^ exhibited a lower *C*_dl_ (26.3 μF cm^–2^) and a higher inhibition efficiency of 95.3% compared with the WEP at the same concentration. WEP-EtOAc at 400 mg L^–1^ showed the best results with a low *C*_dl_ (26.8 μF cm^–2^) and the highest inhibition efficiency (97.0%). This result indicates that the ethyl acetate fraction concentrates the inhibitory molecules.

Figure 7 shows the polarization curves of the MS in HCl solution without and with the extracts WEP and WAP at different concentrations and their fractions at 400 mg L^–1^. Table 5 presents the results obtained from the extrapolation of the Tafel curves. Adding WEP and WAP and their fractions inhibits both anodic and cathodic processes, with the action being much more pronounced in the cathodic process. The OCP generally shifts to more anodic values (Figure 5), whereas the potential corrosion (*E*_corr_) shifts to more cathodic values (Table 6). This result suggests that cathodic polarization influenced the inhibitory action. The inhibition efficiency data derived from *j*_corr_ values align with the results obtained from mass loss measurements (Table 2) and electrochemical impedance results (Table 5 and Figure 6). Again, the ethanolic extract (WEP) presented a higher IE than the aqueous extract (WAP), with the best result obtained with the WEP-EtOAc. With 400 mg L^–1^, the IE was 81.4%, 95.8%, and 81.8% for WEP and its ethyl acetate and residual fractions, respectively. All of these results suggest that the inhibitory molecules were concentrated in the ethyl acetate fraction.

**Figure 7:**
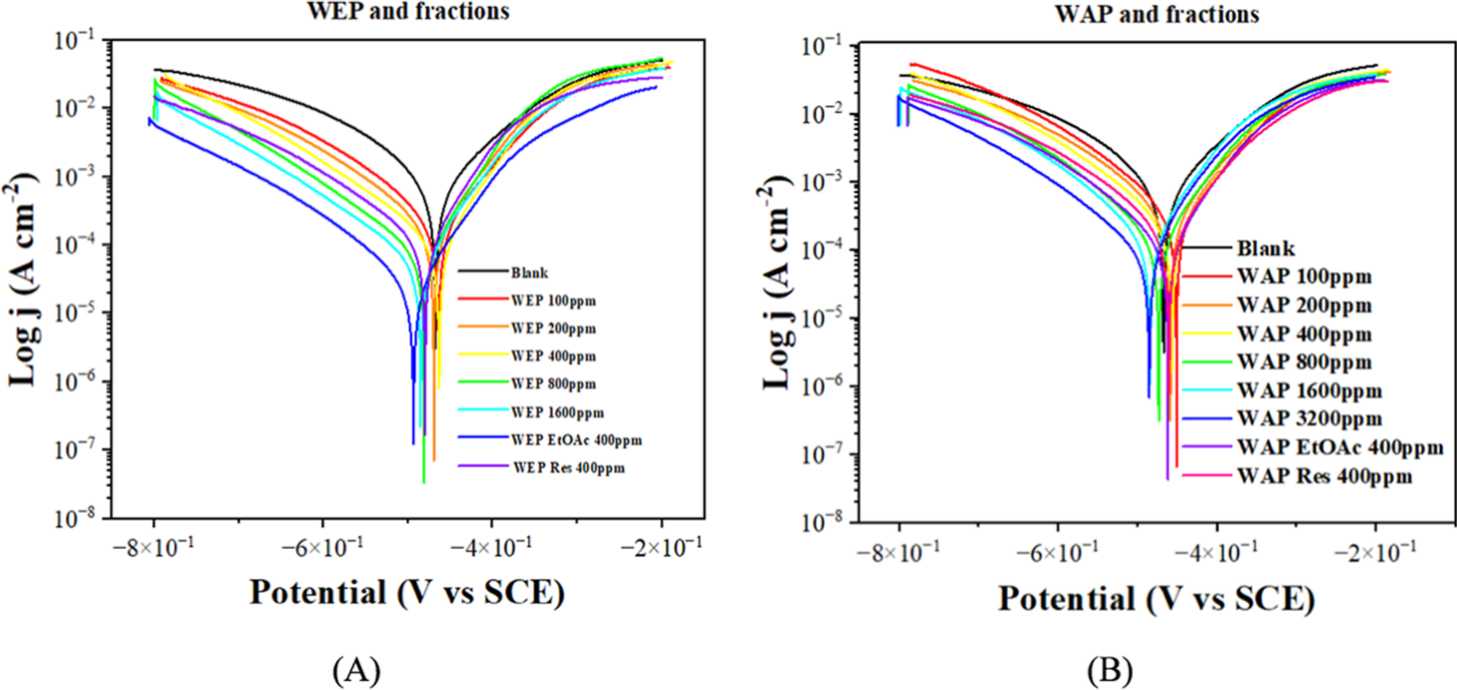
Polarization curves of MS in 1 mol L^–1^ HCl in the absence and presence of WEP (A) and WAP (B) at different concentrations and their ethyl acetate and residual fractions.

**Table 6:** Kinetic Parameters Obtained from Tafel Plots for MS in 1 mol L^–1^ HCl in the Absence and Presence of WEP, WAP, and Their Ethyl Acetate and Residual Fractions

sample	[inhibitor] (mg L^–1^)	OCP (mV/ECS)	*E*_corr_ (mV/ECS)	*j*_corr_ mA cm^–2^	IE (%)
blank	0	–507	–465	0.473	
WEP	100	–491	–458	0.179	62.2
WEP	200	–494	–470	0.139	70.6
WEP	400	–488	–463	0.088	81.4
WEP	800	–501	–481	0.064	86.5
WEP	1600	–496	–485	0.046	90.3
WEP-EtOAc	400	–501	–494	0.020	95.8
WEP-RES	400	–504	–480	0.086	81.8
WAP	100	–486	–451	0.221	53.3
WAP	200	–483	–462	0.176	62.8
WAP	400	–486	–457	0.168	64.5
WAP	800	–490	–474	0.110	76.7
WAP	1600	–494	–485	0.101	78.6
WAP	3200	–502	–486	0.072	84.8
WAP-EtOAc	400	–490	–462	0.083	82.5
WAP-RES	400	–487	–461	0.120	74.6

### 3.5 Scanning Electron Microscopy Integrated with Energy-Dispersive Spectroscopy and X-ray Excited Photoelectron Spectroscopy

Figure 8 displays the MS surface before exposure to the acidic medium, showcasing a smooth texture with noticeable grooves resulting from the abrasion of MS. After the immersion test, the surface of the MS exposed to the acidic medium was significantly altered, exhibiting a rough texture primarily due to the dissolution of iron caused by the acid. However, the extract’s effectiveness became apparent when the immersion test was conducted with the inhibitor present. Although there were some areas of degradation, the surface remained relatively smooth. Energy-dispersive spectroscopy (EDS) analysis revealed that sulfur atoms (0.0065%) are present only in the ethyl acetate fraction of WEP, supporting the idea that glucosinolates were concentrated in this fraction.

**Figure 8:**
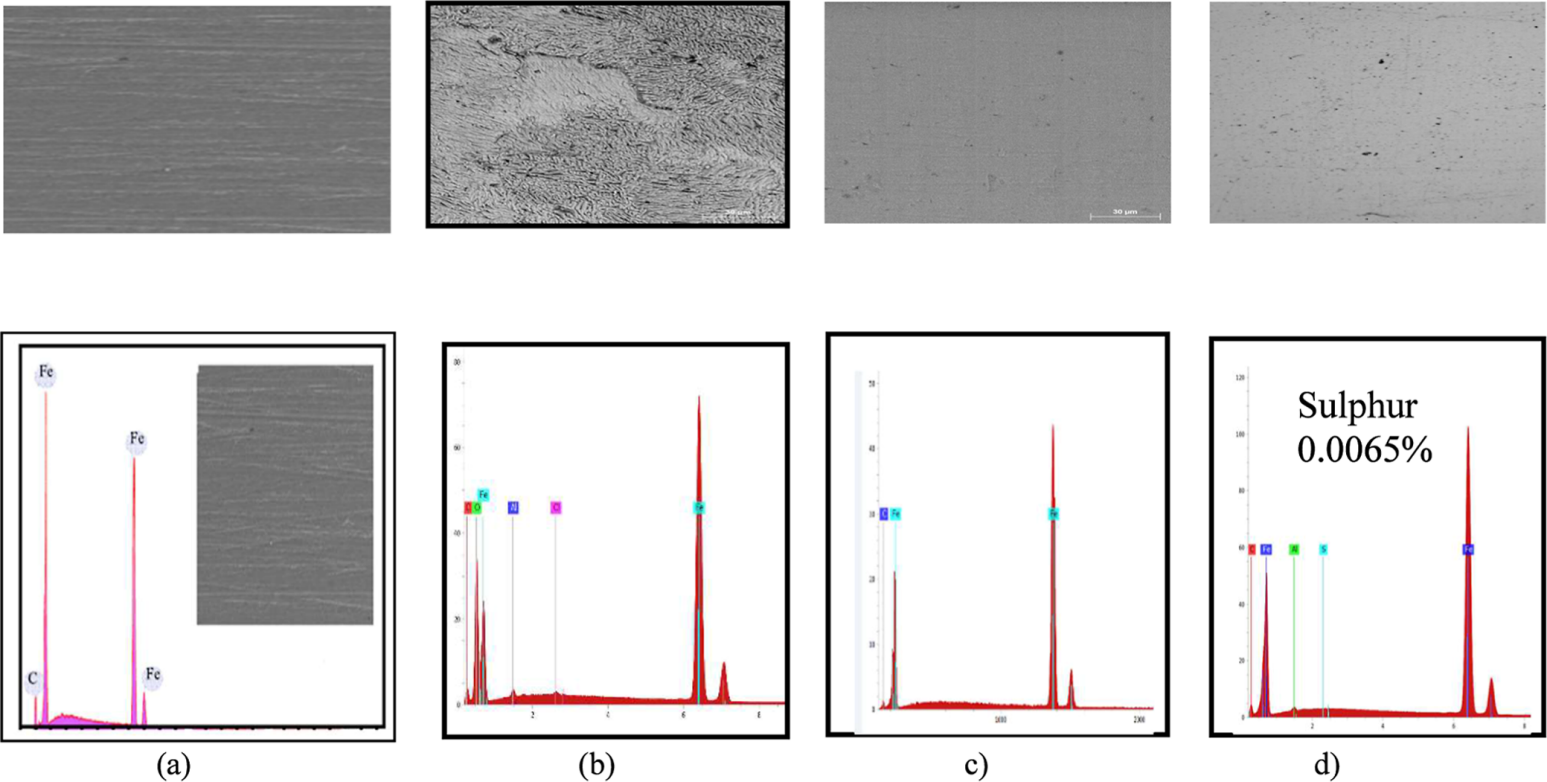
SEM photograph and their respective EDS spectra of the plates before (a) and after exposure to the 1 mol L^–1^ HCl solution without (b) and with WEP (c) and its ethyl acetate fraction with sulfur 0.0065% (d).

The XPS analysis of the MS immersed in a 400 mg L^–1^ solution of WEP-EtOAc (Figure 9a–g) revealed a difference in the sulfur spectrum of WEP (presented in Supporting Information). The signal in the 171 eV region indicated that the interaction between iron and sulfur occurs in the highest oxidation state, specifically as part of a sulfate group. The sulfur detection on the MS surface supports the hypothesis that glucosinolates inhibit MS corrosion in acidic environments. In the Fe 2p spectrum (Figure 9f,g), it is evident that the area representing the presence of nonoxidized metallic iron (Fe°) is more prominent than that observed in the WEP, indicating a higher level of protection for the steel. The XPS spectra of WEP and WAP are presented in the Supporting Information spectrum in Figures S12 and S13.

**Figure 9:**
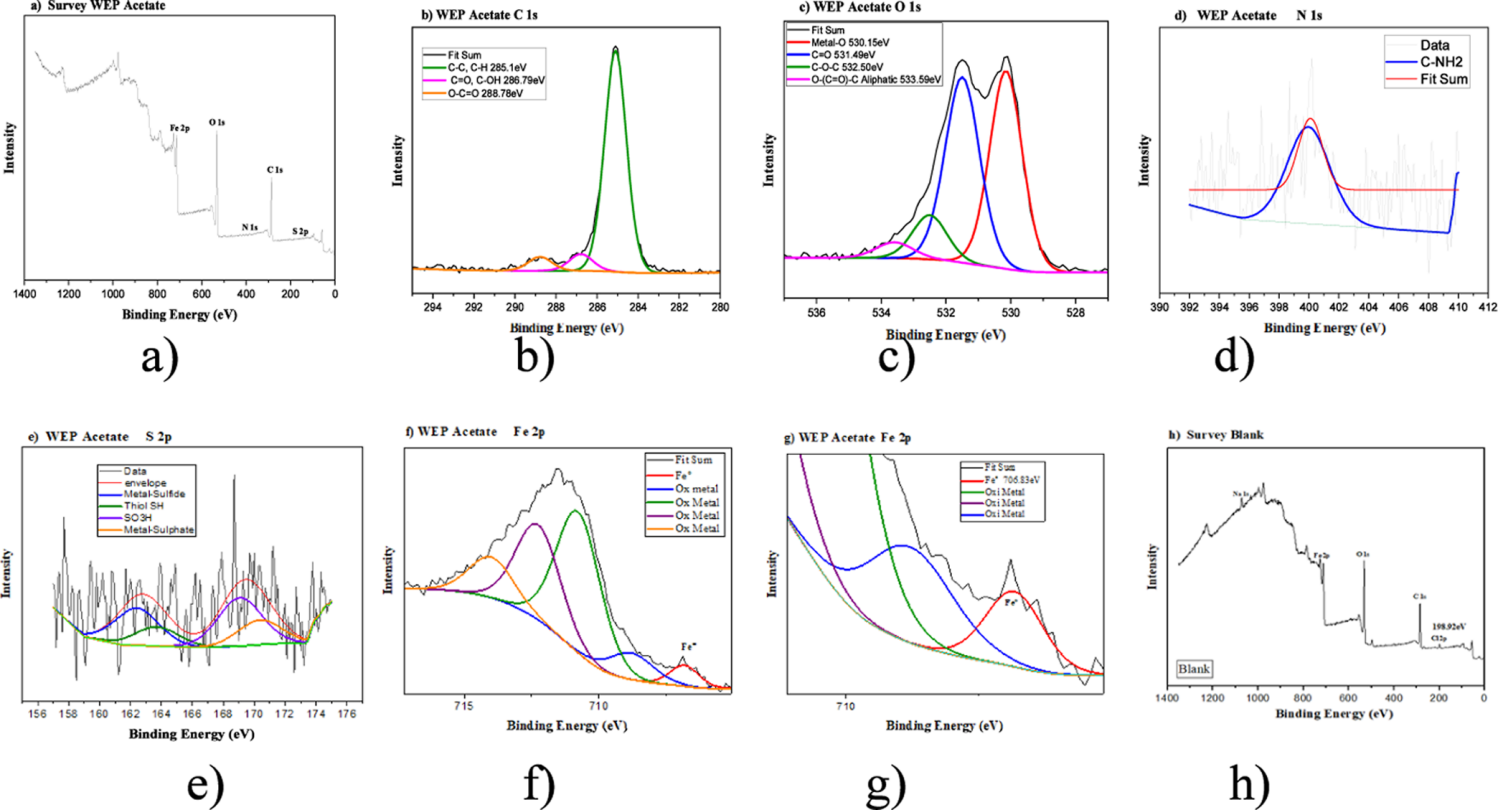
XPS survey spectrum (a) and high-resolution spectra of (b) C 1s, (c) O 1s, (d) N 1s, (e) S 2p, (f) Fe 2p, and (g) Fe 2p (magnified) of MS in the absence and presence of 400 mg L^–1^ of ethyl acetate fraction of WEP in HCl 1 mol L^–1^.

### 3.6 MALDI-TOF-MS and MALDI-FT-ICR-MS Analyses for the Acetate Fraction of WEP

Figure 10 shows the MALDI-TOF-FT-MS(±) spectra for a MS surface sample after two hours of immersion in 1 mol L^–1^ HCl containing 400 mg L^–1^ WEP-AcOEt, using three different methods: (a) unwashed, (b) scraped with a metal spatula and washed with double distilled water, and (c) the solution formed by scraping and washing the MS plate (from method b). The spectra obtained by these different methods indicated the presence of glucosinolates on the MS surface. Despite the variations in the number of signals, this demonstrates the efficient ionization of these molecules by the MALDI source in both positive and negative modes. The presence or absence of 2,5-dihydroxybenzoic acid (DHB) as a matrix did not affect the detection of the target molecules.

**Figure 10:**
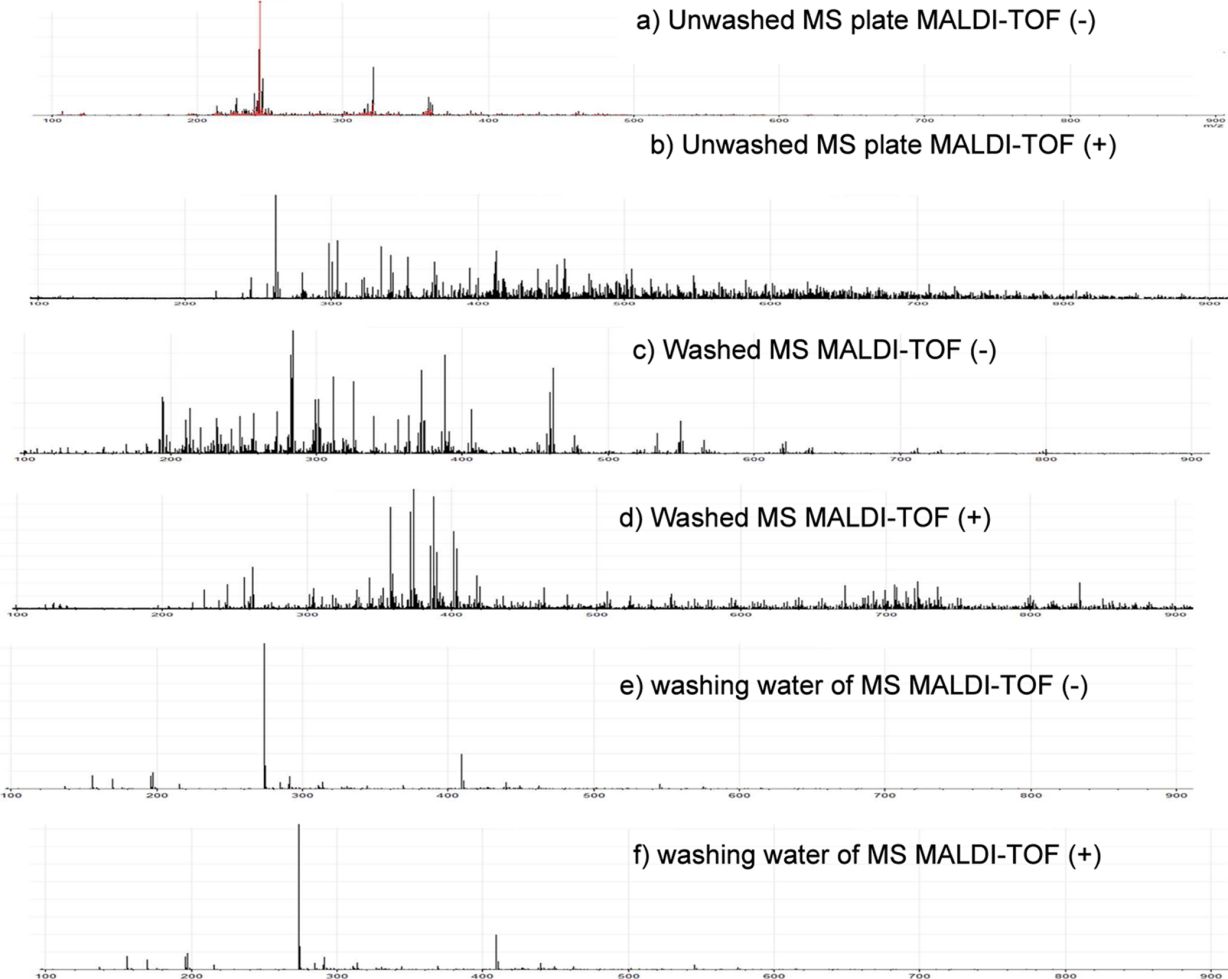
MALDI-TOF­(±) spectra with and without (2,5-dihydroxybenzoic acid) matrix, obtained with the MS plate (test specimen) with and without washing, fixed directly to the equipment, and from the solution obtained by washing the plate. Figures a (−) mode and b (+) mode show the spectra obtained from the MS plate immersed in a 1 mol L^–1^ HCl solution containing 400 mg L^–1^ of WEP-EtOAc for 2 h without washing and drying with hot air. Figures c (−) and d (+) show the spectra of the scraped and washed MS plate. Figures e and f show spectra obtained through the analysis performed using the water from washing the plate in positive and negative modes.

The obtained signals from MALDI-TOF-FT-MS­(+) were compared with literature data on the secondary metabolites of *A. rusticana*, including glucosinolates and isothiocyanates, as well as terpenes, polyphenols, flavonoids, and various organic acids, such as ferulic, gallic, 4-hydroxybenzoic, malic, citric, and sinapinic acids. According to the literature,^13^ the interpretation primarily focused on glucosinolates, which are particularly interesting due to their chemical structure. This structure includes heteroatoms like oxygen, nitrogen, and sulfur, which are significant for their corrosion-inhibiting properties.^13,21,49,51,52^ The FT-MS-MALDI-TOF spectrum obtained in positive mode shows multiple signals in the *m*/*z* range 100–600. Accurate mass measurements can more reliably identify the chemical profile of glucosinolate species in the WEP-EtOAc. The chemical profile from this fraction reveals three distinct sets of ions in specific spectrum regions: (i) In the *m*/*z* 90–300 range, isothiocyanates such as phenylisothiocyanate are annotated at *m*/*z* 136.0354 [C_7_H_6_NS]^+^. (ii) The *m*/*z* range 300–500 preferentially concentrates glucosinolates, such as glucoputranjivin, annotated at *m*/*z* 360.0919 [C_10_H_18_NO_9_S_2_]^−^. (iii) In the *m*/*z* range 500–900, dimers and various glycosides.^51^ These include the significant glucosinolates, glucoraphanin (C_12_H_23_NO_10_S_3_), and glucoibarin (C_15_H_29_NO_10_S_3_), assigned as protonated molecules at *m*/*z* = 438.0584 Da and *m*/*z* = 480.0929 Da, respectively. The peak *m*/*z* 449.0510 (error = −0.0173 Da) exhibits a degree of unsaturation (DBE) of 8 and was assigned to the [M + H]^+^ for glucobrassicin (Figure 11). The results obtained using DHB as a matrix in the MALDI-TOF-FT-MS­(+) spectra of the WEP-EtOAc, as shown in Figure 10a–f, can be compared for the protonation of molecules.^13,21,53,54^

**Figure 11:**
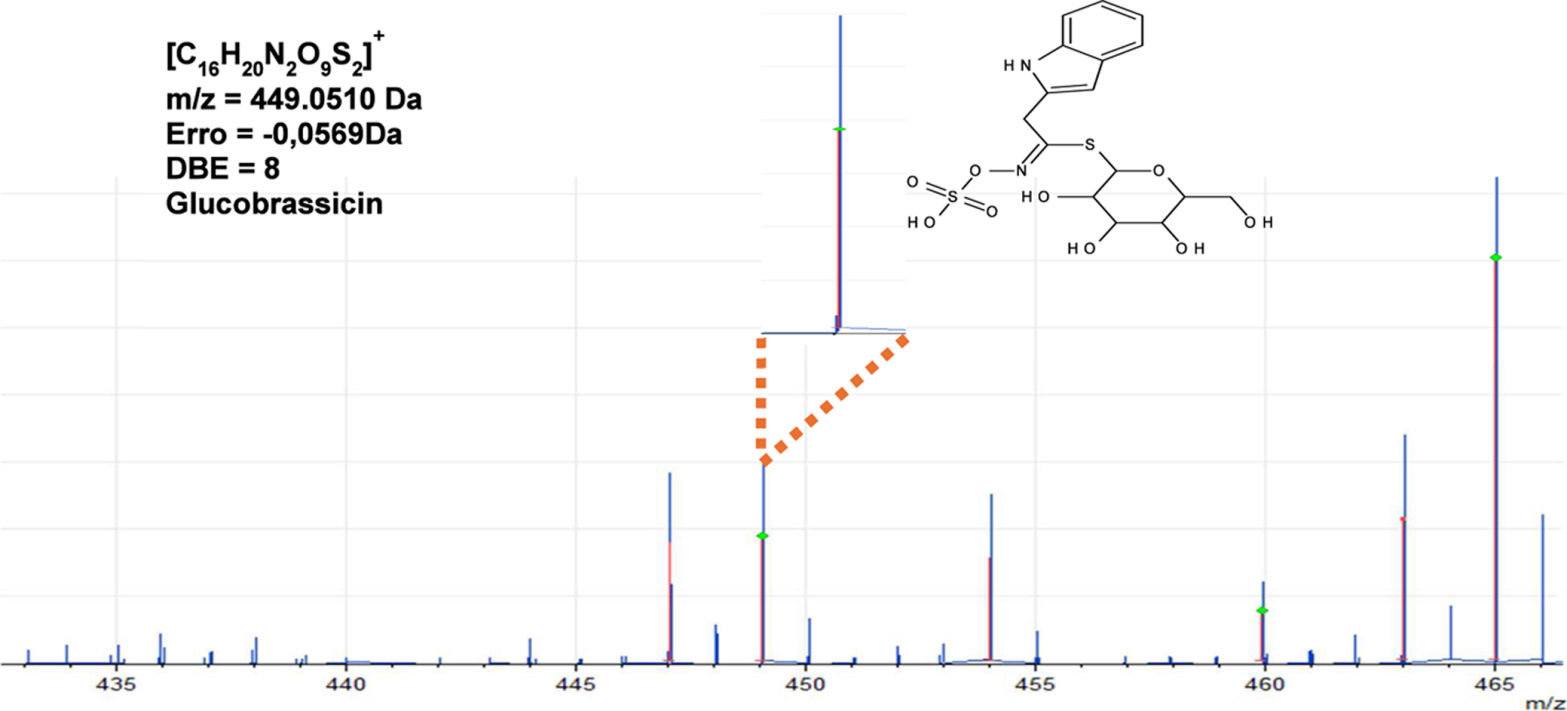
MALDI-TOF-MS­(+) spectrum with magnification of the 449 signal, glucobrassicin.

Different chemical substances were identified on the surface of the washed and unwashed steel plate after 2 h of immersion in HCl and by analyzing the solution generated by the washing water. Table 7 shows the glucosinolates identified (chemical structures of phytochemicals are shown in Supporting Information, Figure S14 and Table S6) by MALDI-TOF-MS(±) and MALDI-FT-ICR-MS(±) in agreement with the literature. Figure 11 shows an enlarged signal of glucosinolate glucobrassicin.

**Table 7:** Proposed Structures of Possible Glucosinolate and Isothiocyanate Molecules Adsorbed on the Surface of the Unwashed, Washed, and Plate Wash Water by MALDI-FT-ICR-MS(±)/MALDI-TOF-MS(±)/MALDI-TOF-MSMS(±)

propose structures identified in the WEP-EtOAc	ion [M – H]^−^/[M + H]^+^	*m*/*z*_teórico_ Da	*m*/*z*_exp_ Da	error Da	DBE
sinigrim	[C_10_H_17_NO_9_S_2_ – H]^−^	358.0272 Da	358.0336 Da ms/ms	0.0064 Da	3
			313.9754 Da		
			314.9821 Da		
			315.9861 Da		
			316.9827 Da		
			290.9978 Da		
			277.9136 Da		
			274.9649 Da		
			259.0119 Da		
			138.9707 Da		
			120.0737 Da		
			98.9762 Da		
glucoputranjivin or propyl-glucosinolate	[C_10_H_20_NO_9_S_2_ + H]^+^	362.0574 Da	361.9739 Da	–0.0835 Da	2
	[C_10_H_19_NO_9_S_2_ – H]^−^	360.0428 Da	359.9951 Da	–0.0477 Da	2
			361.9739 Da ms/ms		
			317.9858 Da		
			282.0061 Da		
gluconapin	[C_11_H_19_NO_9_S_2_ – H]^−^	372.0428 Da	372.0402 Da	–0.0026 Da	3
glucocochlearin	[C_11_H_21_NO_9_S_2_ + H]^+^	376.0730 Da	375.9756 Da	–0.0975 Da	2
	[C_11_H_21_NO_9_S_2_ – H]^−^	374.0585 Da	374.0567 Da	–0.0018 Da	
7-carboxy-methyl-glucosinolate	[C_9_H_15_NO_11_S_2_ – H]^−^	376.0014 Da	376.0023 Da	–0.0009 Da	3
glucokolrabiin or glucojiaputin	[C_12_H_23_NO_9_S_2_ – H]^−^	388.0741 Da	388.0714 Da	–0.0016 Da	2
glucobrassicanapin	[C_12_H_21_NO_9_S_2_ + H]^+^	388.0730 Da	388.0367 Da	–0.0364 Da	3
glucorapiferin/epi-progoitrin	[C_11_H_19_NO_10_S_2_ – H]^−^	388.0378 Da	388.0440 Da	0.0062 Da	3
glucoconringiin or glucocappariflexin (3,4,5-trihydroxy-6-(hydroxymethyl)tetrahydro-2*H*-pyran-2-yl) (1*E*)-4-hydroxy-*N*-(sulfooxy)pentanimidothioate	[C_11_H_21_NO_10_S_2_ – H]^−^	390.0534 Da	390.0368 Da	–0.0166 Da	2
glucotropaeolin	[C_14_H_19_NO_9_S_2_ – H]^−^	407.0428 Da	407.0375 Da	–0.0053 Da	6
glucoiberin	[C_11_H_22_NO_10_S_3_ – H]^−^	422.0255 Da	422.0164 Da	–0.0091 Da	2
			ms/ms		
			406.9861 Da		
			358.9497 Da		
			316.9754 Da		
			290.9048 Da		
			274.9649 Da		
			258.9700 Da		
			227.0350 Da		
			195.0452 Da		
gluconasturtiin (phenethylglucosinolate)	[C_15_H_21_NO_9_S_2_ – H]^−^	422.0585 Da	422.0567 Da	–0.0018 Da	3
glucoraphanin/4-methylsulfinylbutylglucosinolate	[C_12_H_24_NO_10_S_3_ + H]^+^	438.0557 Da	438.0584 Da	0.0027 Da	2
	[C_12_H_23_NO_10_S_3_ – H]^−^	436.0411 Da	436.0403 Da	–0.0008 Da	
glucobarbarin/epiglucobarbarin 3,4,5-trihydroxy-6-(hydroxymethyl)tetrahydro-2*H*-pyran-2-yl(1*Z*)-2-(3-methoxyphenyl)-*N*-(sulfooxy)ethanimidothioate	[C_15_H_21_NO_10_S_2_ + H]^+^	440.0680 Da	440.0741 Da	0.0061 Da	6
nonylglucosinolate	[C_16_H_31_NO_9_S_2_ + H]^+^	446.1513 Da	446.1110 Da	–0.0403 Da	2
	[C_16_H_31_NO_9_S_2_ – H]^−^	444.1367 Da	444.1418 Da	0.0051 Da	
glucobrassicin	[C_16_H_20_N_2_O_9_S_2_ + H]^+^	449.0683 Da	449.0510 Da	–0.0173 Da	8
	[C_16_H_19_N_2_O_9_S_2_ – H]^−^	447.0537 Da	447.0421 Da	–0.0116 Da	
naphthylmethylglucosinolate	[C_18_H_21_NO_9_S_2_ + H]^+^	460.0730 Da	459.9915 Da	–0.0815 Da	9
7-carboxy-methyl-glucosinolate	[C_15_H_27_NO_11_S_2_ – H]^−^	460.0953 Da	460.0617 Da	–0.0336 Da	3
5-hydroxyglucobrassicin/4-hydroxyglucobrassicin	[C_16_H_20_N_2_O_10_S_2_ + H]^+^	465.0632 Da	465.0572 Da	–0.0509 Da	8
4-methoxyglucobrassicin neoglucobrassicin	[C_17_H_22_N_2_O_10_S_2_ + H]^+^	479.0789 Da	479.0712 Da	–0.0077 Da	8
brassicin	[C_22_H_22_O_12_ + H]^+^	479.1184 Da	479.1012 Da	–0.0172 Da	12
glucoibarin	[C_15_H_29_NO_10_S_3_ + H]^+^	480.1026 Da	480.0929 Da	–0.0097 Da	2

The surface analysis of MS conducted using MALDI-TOF-MS and MS/MS (Figures S15 and S16, MS/MS of ions 358 and 422, negative mode, Supporting Information) suggests the presence of glucosinolates adsorbed onto the plate. These compounds’ heteroatoms (O, S, and N) adsorb to the metal surface, forming a protective layer that helps mitigate corrosion.^55^ Therefore, these glucosinolates can donate electrons to the metal surface, mitigating the corrosion process.^39,56,57^ The partitioning performed in ascending order of polarity concentrated the glucosinolate chemical group in the ethyl acetate fraction. Based on these results, it can be inferred that these compounds (glucosinolates) are likely responsible for the significant enhancement in the inhibition efficiency (IE) of the *A. rusticana* ethanolic extract. When partitioned with ethyl acetate, the IE increased during the 2 h mass loss test at 400 ppm from 85.0% to 97.5% (Table 2). A similar improvement was observed from 86.5% to 97.0% according to electrochemical impedance data (Table 5), and from 81.4% to 95.8% based on polarization curve results (Table 6). Table S2 (presented in Supporting Information) shows the importance of the partition performed with the crude extracts. It is possible to relate the inhibition efficiency to certain existing chemical groups. WEP showed the highest inhibition efficiency among the crude extracts. The chemical profile of WEP, traced by FTIR, ESI-MS(±), and MALDI-TOF-MS(±), showed the presence of aldehydes, ketones, amino acids, hydrocarbons, terpenes, flavonoids, furans, organic acids, fatty acids, phenolic acids, flavonoids, tannins, coumarins, isothiocyanates, and glucosinolates. The hexane fraction removed the hydrocarbons, terpenes, fatty acids, some organic acids, aldehydes, isothiocyanates, phenolic acids, and coumarins. The ethyl acetate fraction concentrated the glucosinolates, amino acids, flavonoids, tannins, and some aldehydes and isothiocyanates, which were not extracted by hexane. The residual fraction comprises organic acids, phenolic acids, furans, isothiocyanates, coumarins, ketones, aldehydes, and flavonoids.^22^ WAP did not present the expressive presence of glucosinolates, which were extracted by hexane, leaving their ethyl acetate and residual fractions without this group and with the lowest IE among the fractions. The results show a high possibility that the glucosinolate group primarily controls the inhibition efficiency. Tables S1 and S3 (presented in Supporting Information) show the results obtained by mass spectrometry, [M – H]^−^, [M + H]^+^, *m*/*z*, error (Da), and possible chemical structures according to ref 22. Tables S2 and S4 show the partition of the ethanolic extract of *A. rusticana*, indicating the separation of substances by polarity. The glucosinolates were concentrated in the ethyl acetate fraction, which leads us to believe that the high inhibition potential of this fraction is due to the various molecules belonging to the glucosinolate group, which were not identified in the hexane and residual fractions.

Finally, Table 8 compares the anticorrosive efficiency of the studied extract with those of other inhibitors reported in the literature. The ethyl acetate fraction of WEP, together with the pineapple stem extract, exhibited the highest inhibition efficiency against MS corrosion in HCl solution. In addition, the ethanolic extract of *A. rusticana* showed 93.3% inhibition efficiency in a sulfuric acid solution. These findings highlight the potential of *A. rusticana* as an effective corrosion inhibitor for MS in acidic media.

**Table 8:** Comparison of the Corrosion Inhibition Efficiency of the Studied Extract and Reported Inhibitors on MS in Acidic Medium

inhibitor	[inhibitor]	corrosive medium	IE %	references
*Armoracia rusticana* ethanolic extract	100 mg/L	0.5 M H_2_SO_4_	93.3	^49^
*Lycoris radiata* and *Lycoris chinensis* leaf extract	3%	5% HCl	91.5	^58^
pineapple stem aqueous extract (Bromelain)	1000 mg/L	1 M HCl	97.6%	^59^
*Ficus tikoua* leaves aqueous extract	200 mg/L	1 M HCl	95.8%	^60^
*Luffa cylindrica* leaf ethanolic extract	2000 mg/L	1 M HCl	88.0%	^61^
roasted coffee aqueous extracts	1000 mg/L	1 M HCl	94.0%	^6^
ethyl acetate fraction of WEP (this study)	400 mg/L	1 M HCl	97.5%	

## 4 Conclusions

The study investigated the corrosion-inhibitory effects of ethanolic and aqueous extracts of *A. rusticana* and their fractions on MS corrosion in HCl solution. The partitioning of the crude extracts, both aqueous and ethanolic, was performed in the ascending order of polarity, using hexane and ethyl acetate as solvents. This process revealed differences in inhibition efficiency among the fractions. Notably, the fractions with a higher concentration of glucosinolates demonstrated greater inhibitory effectiveness. WEP exhibited inhibition efficiency exceeding 85% from 400 mg L^–1^ after 2 h of immersion in an acidic medium. The ethyl acetate fraction of WEP achieved an inhibition efficiency of over 97% at a concentration of 400 mg L^–1^, classifying it as an excellent green inhibitor. Furthermore, the inhibitor’s stability was demonstrated over 24 h, presenting an IE constant with time with little increase. The electrochemical impedance diagrams showed an inductive loop in the low-frequency range with a high inhibitor concentration, which indicates a change in the corrosion mechanism. The polarization data calculated by the Tafel extrapolation method showed that *E*_corr_ shifts to more negative values. In contrast, the OCP shifts to more positive values, indicating that the cathodic polarization influences the inhibitory action.

The analysis of MALDI-MS conducted by applying the laser directly to a metal plate demonstrated a new application of this technique for surface analysis in corrosion studies. By fitting the plate inside the device, the adsorbed molecules received direct exposure to the laser, resulting in the formation of ions on the metal surface. These ions were analyzed using ion cyclotron resonance (ICR) and time-of-flight (TOF), providing high resolution and efficiency. As a result, various structures of glucosinolates were proposed, which were present on the metal plate after 2 h of immersion in 1 mol L^–1^ HCl. The unwashed plate exhibited a higher quantity of adsorbed molecules. This analysis proved effective even without applying a matrix to assist in ionization, successfully identifying adsorbed molecules after the steel plate.

EDS and XPS analyses revealed that sulfur atoms interact with iron on the surface of MS. This supports the idea that glucosinolates are significant phytochemicals that can be adsorbed onto the metal surface, helping to prevent the dissolution of iron in acidic environments.

Although the Langmuir isotherm provided the best fit for the experimental results among all the isotherms, the MALDI-MS technique revealed the presence of different molecules, such as glucosinolates, isothiocyanates, terpenes, polyphenols, flavonoids, and various organic acids, on the steel surface, which is not consistent with the premises of this model. Therefore, other isotherms should be considered, as they fit the experimental results well. This includes repulsive interactions between the adsorbed molecules or the adsorption of bulky molecules such as glucosinolates.

## Supplementary Material



## Notes

FTIR of WAP and fractions, ESI-MS(±) of ethanolic extract of the *A. rusticana*—WEP, ESI-MS­(±) of hexane fraction of ethanolic extract of the *A. rusticana*—WEP hexane, ESI-MS(±) of ethyl acetate fraction of ethanolic extract of the *A. rusticana*—WEP acetate, ESI-MS(±) of residual fraction of ethanolic extract of the *A. rusticana*—WEP residual, ESI-MS(±) of aqueous extract of the *A. rusticana*—WAP, ESI-MS(±) of hexane fraction of aqueous extract of the *A. rusticana*—WAP hexane, ESI-MS(±) of ethyl acetate fraction of aqueous extract of the *A. rusticana*—WAP acetate, ESI-MS(±) of residual fraction of aqueous extract of the *A. rusticana*—WAP residual, isotherms of Langmuir, Temkin, Flory–Huggins and El-Awady of the WEP, isotherms of Langmuir, Temkin, Flory–Huggins and El-Awady of the WAP, XPS spectrum of WEP (ethanolic extract of the *A. rusticana*), XPS spectrum of WAP (aqueous extract of the *A. rusticana*), possible chemical structures of molecules adsorbed on the MS surface, identified by mass spectrometry MALDI-ICR-MS(±) and MALDI-TOF-MS(±), proposal for fragmentation (MS/MS (−)) of glucobrassicin glucosinolate (422 Da), proposal for fragmentation (MS/MS (−)) of sinigrin glucosinolate (358 Da), ESI-QTOF/ESI-FTICR/MALDI-FTICR/MALDI-TOF of the ethanolic extract of *A. rusticana*—WEP—and fractions (hexane, ethyl acetate and residual), partition of ethanolic extract of *A. rusticana*—WEP and fractions (hexane, ethyl acetate and residual), ESI-QTOF/ESI-FTICR/MALDI-FTICR/MALDI-TOF of aqueous extract of *A. rusticana*—WAP and fractions (hexane, ethyl acetate, and residual), partition of aqueous extract of *A. rusticana*—WAP and fractions (hexane, ethyl acetate and residual), Langmuir, Temkin, Flory–Huggins, and El-Awady isotherms equations of WEP and WAP, and proposed structures of molecules possibly adsorbed on the surfaces of unwashed and washed plates, as well as in the wash water, identified by MALDI-FT-ICR-MS(±), MALDI-TOF-MS(±), and MALDI-TOF-MS/MS(±) (PDF)
