# Supplementary material for: Study of Extracts of A. rusticana as Corrosion Inhibitors of Mild Steel in 1 mol L–1 HCl Solution Using MALDI-TOF-MS
Source: ACS Omega. 2025 Oct 31;10(44):53122–37. doi: 10.1021/acsomega.5c07723 (PMC12613137; doi:10.1021/acsomega.5c07723)
Supplement: Supplementary file 1 [file ao5c07723_si_001.pdf]

"Study of extracts of A. rusticana as corrosion inhibitors of mild steel in 1 mol L^{-1} HCl solution using MALDI-TOF-MS

Iendel Rubio do Nascimento[1*], Klícia Carla de Santana de Lima[2], Lidilhone Hamerski[3], and Eliane D'Elia[1*]

1. Universidade Federal do Rio de Janeiro, Instituto de Química, Avenida Athos da Silveira Ramos, 149, 21941-909, Rio de Janeiro, Brazil;

2. Universidade Federal do Rio de Janeiro, Campus Duque de Caxias, Rodovia Washington Luiz, 19.593, km 104,5, 25.240-005, Duque de Caxias, Brazil;

3. Universidade Federal do Rio de Janeiro, Instituto de Pesquisas de Produtos Naturais Carlos Chagas Filho - IPPN, 373, 21941-853, Rio de Janeiro, Brazil;

Corresponding authors: eliane@iq.ufrj.br and iendelrubio@gmail.com

SUPPLEMENTARY MATERIAL

S1: FTIR of WAP and Fractions

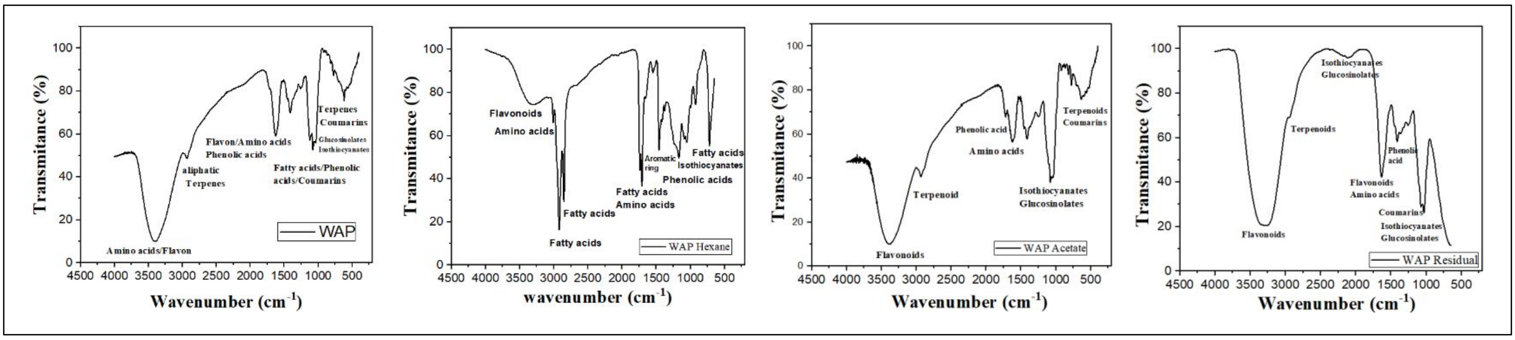

S2: ESI-MS (±) of ETHANOLIC EXTRACT of the *Armoracia rusticana* – WEP

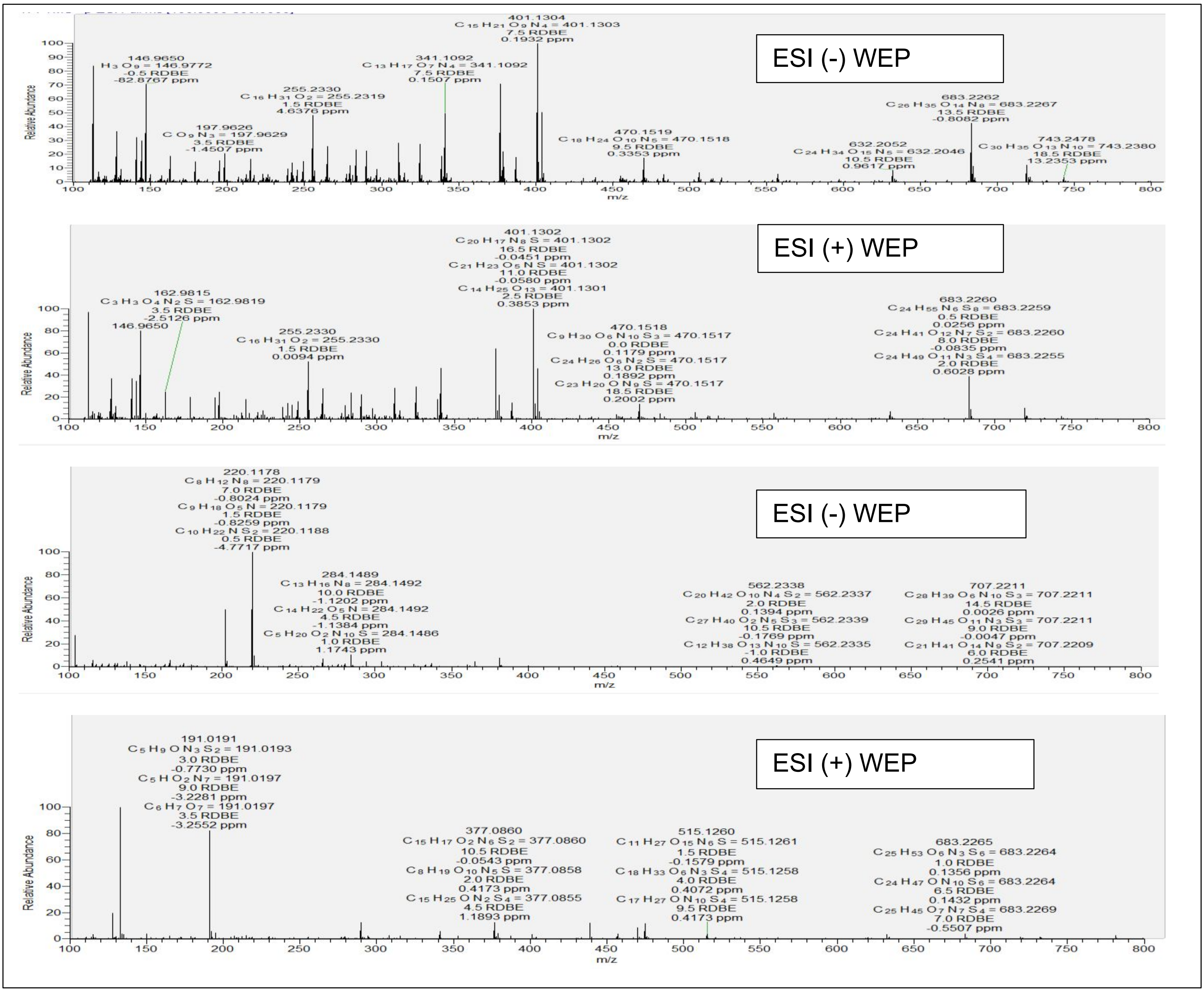

S3: ESI-MS (±) of Hexane fraction of ETHANOLIC EXTRACT of the *Armoracia rusticana* -WEP Hexane

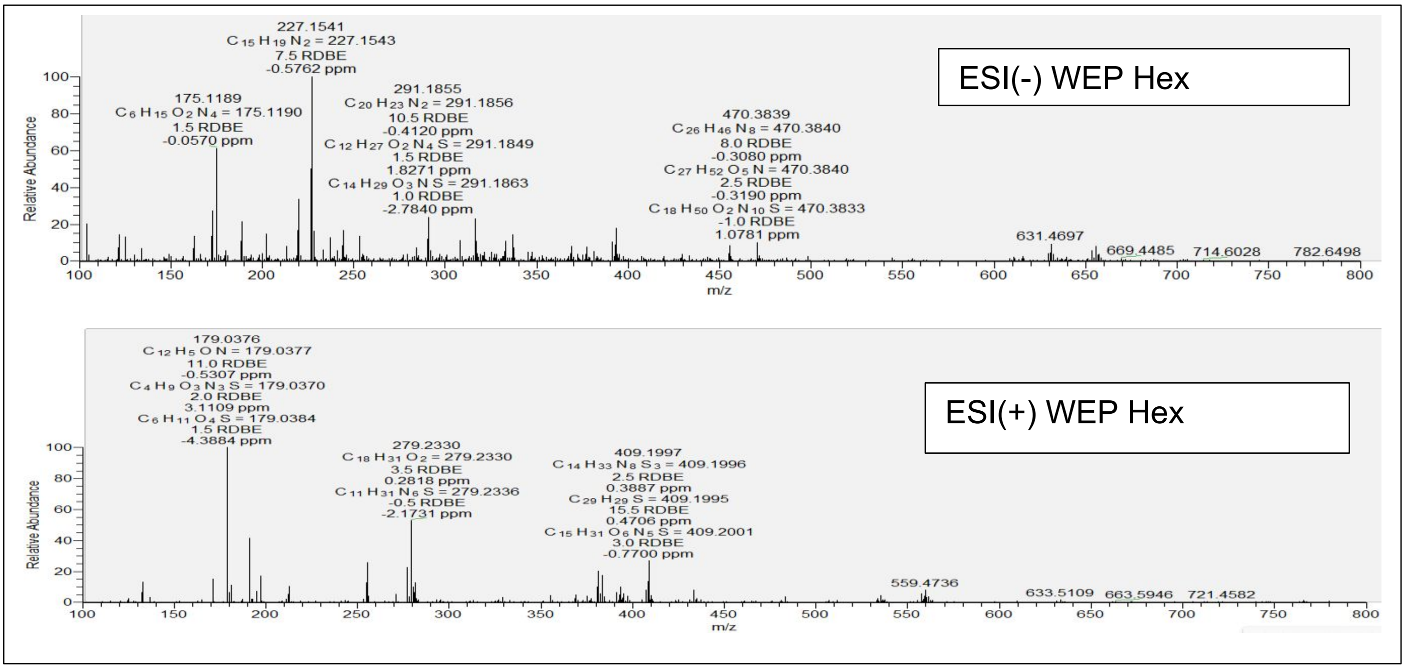

S4: ESI-MS (±) of Ethyl Acetate fraction of ETHANOLIC EXTRACT of the *Armoracia rusticana* - WEP Acetate

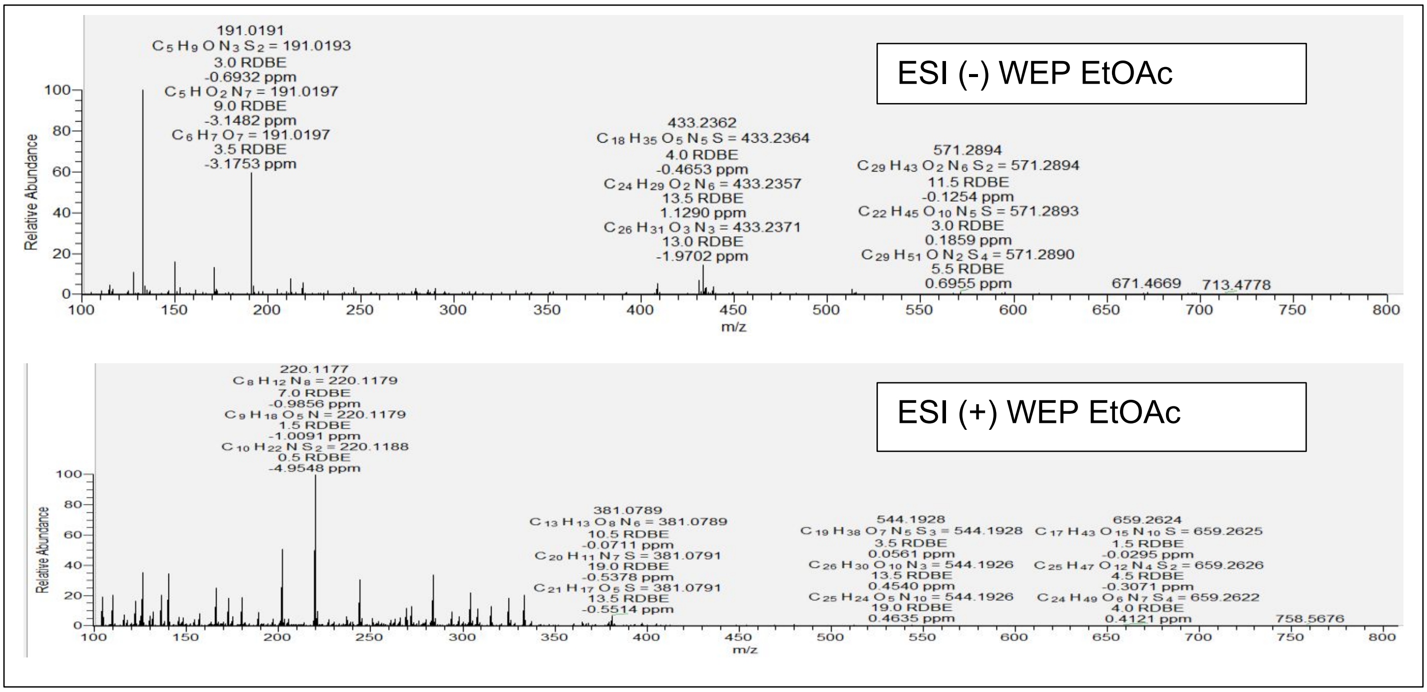

S5: ESI-MS (±) of residual fraction of ETHANOLIC EXTRACT of the *Armoracia rusticana* - WEP Residual

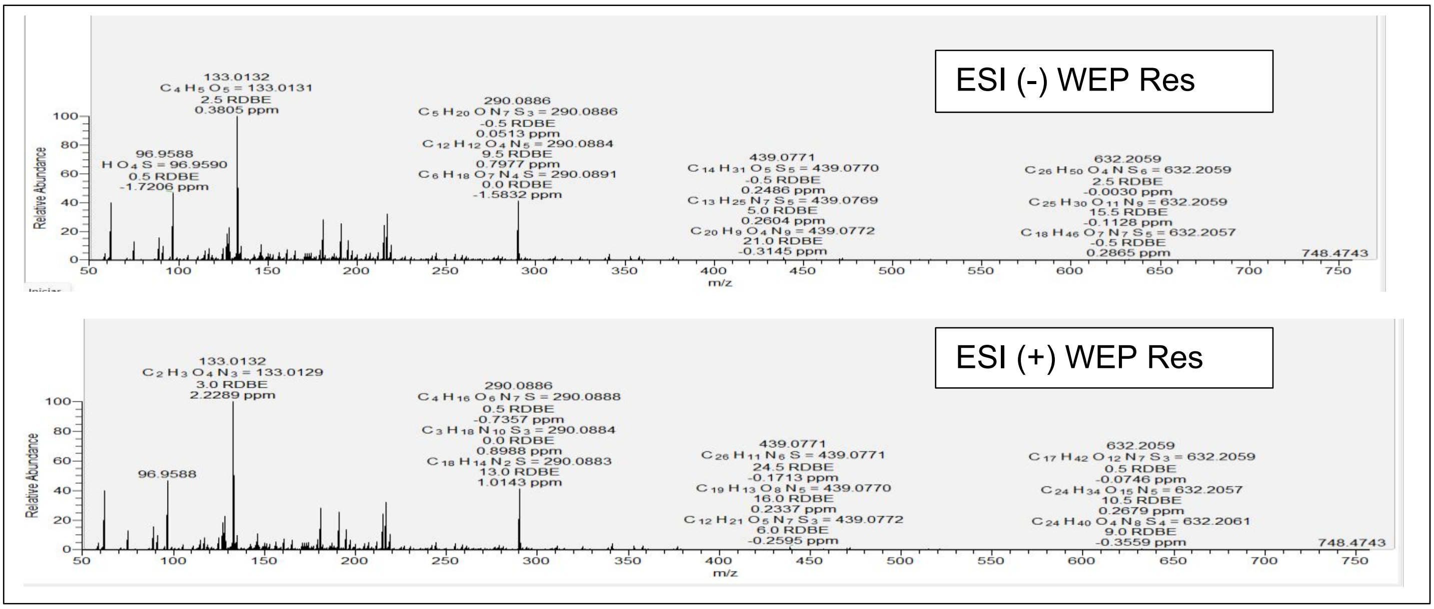

S6: ESI-MS (±) of AQUEOUS EXTRACT of the *Armoracia rusticana* - WAP

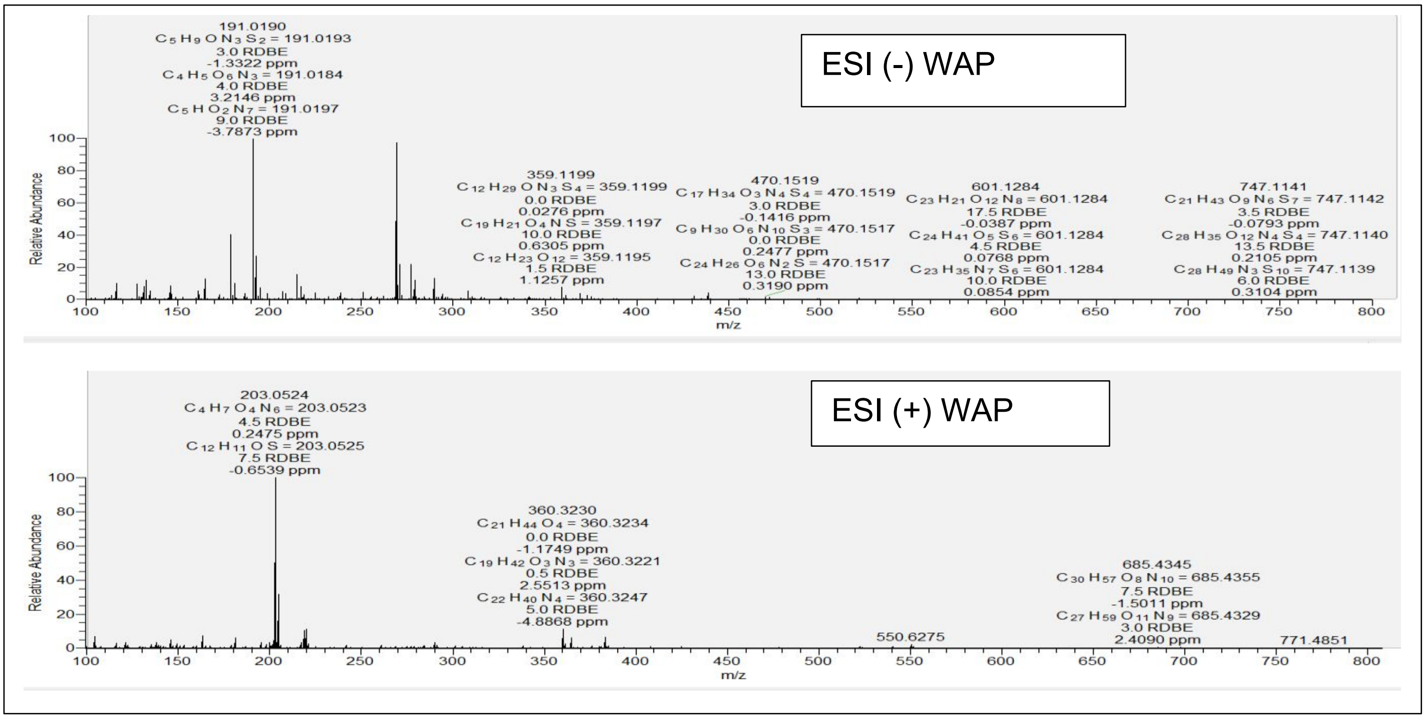

S7: ESI-MS (±) of Hexane fraction of AQUEOUS EXTRACT of the *Armoracia rusticana* – WAP Hexane

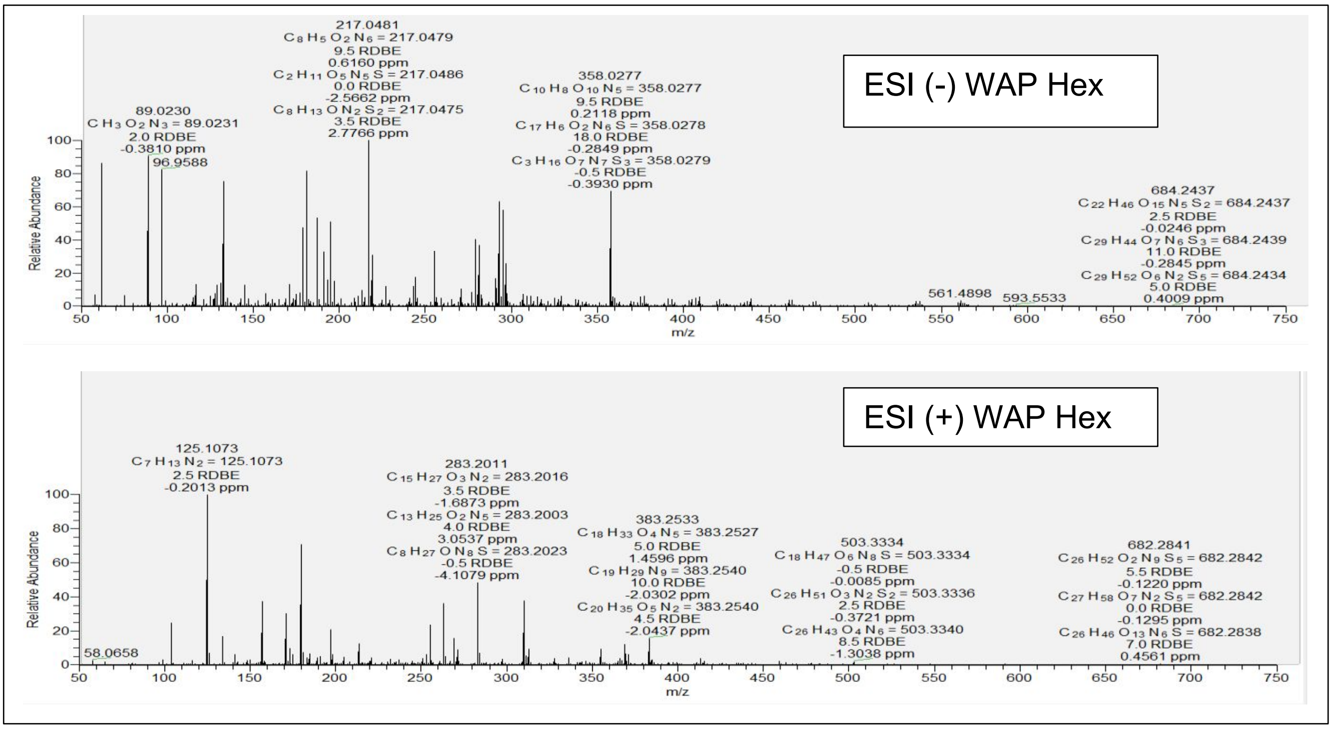

S8: ESI-MS (±) of Ethyl Acetate fraction of AQUEOUS EXTRACT of the *Armoracia rusticana* – WAP Acetate

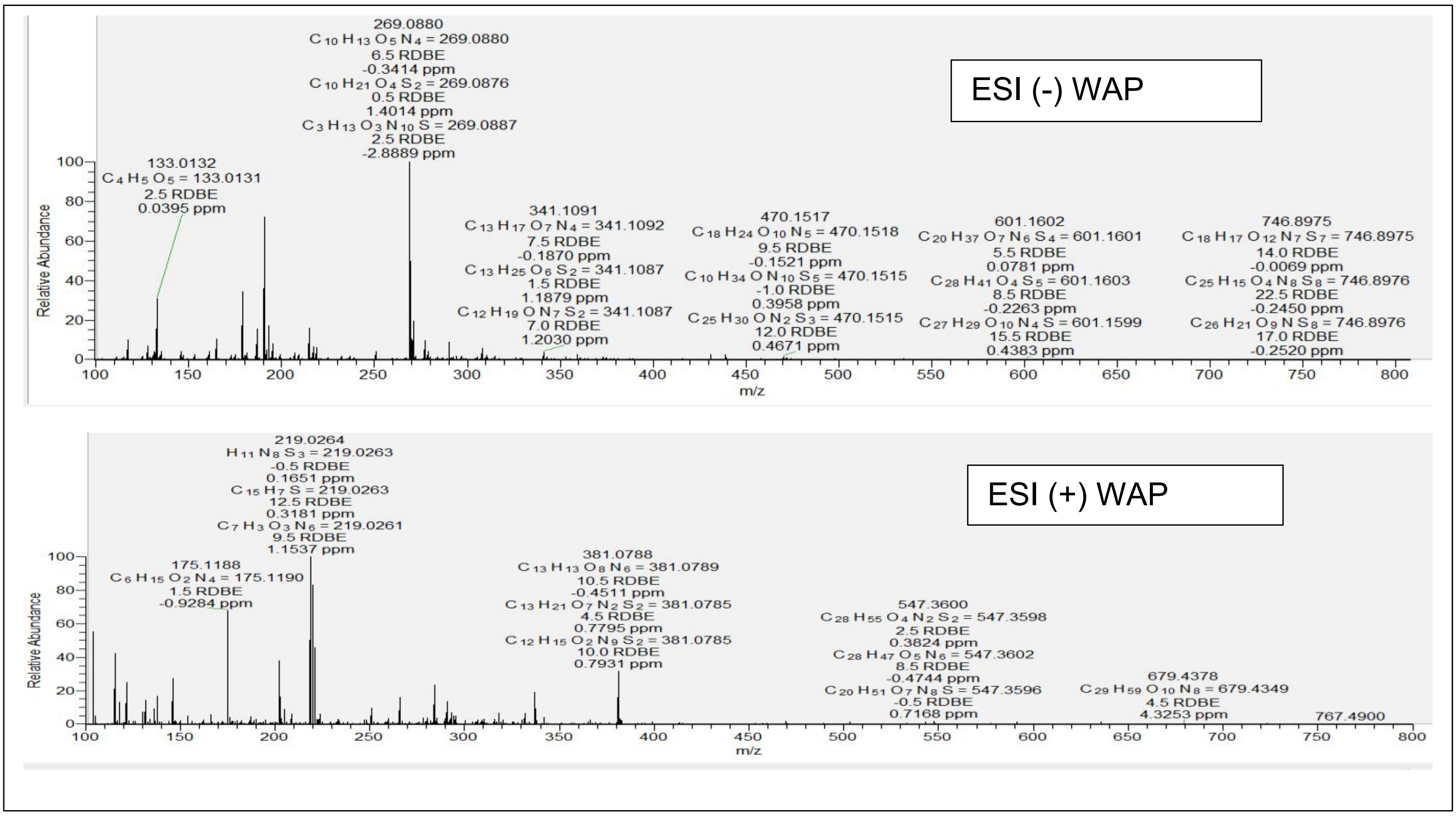

S9: ESI-MS (±) of residual fraction of AQUEOUS EXTRACT of the *Armoracia rusticana* – WAP Residual

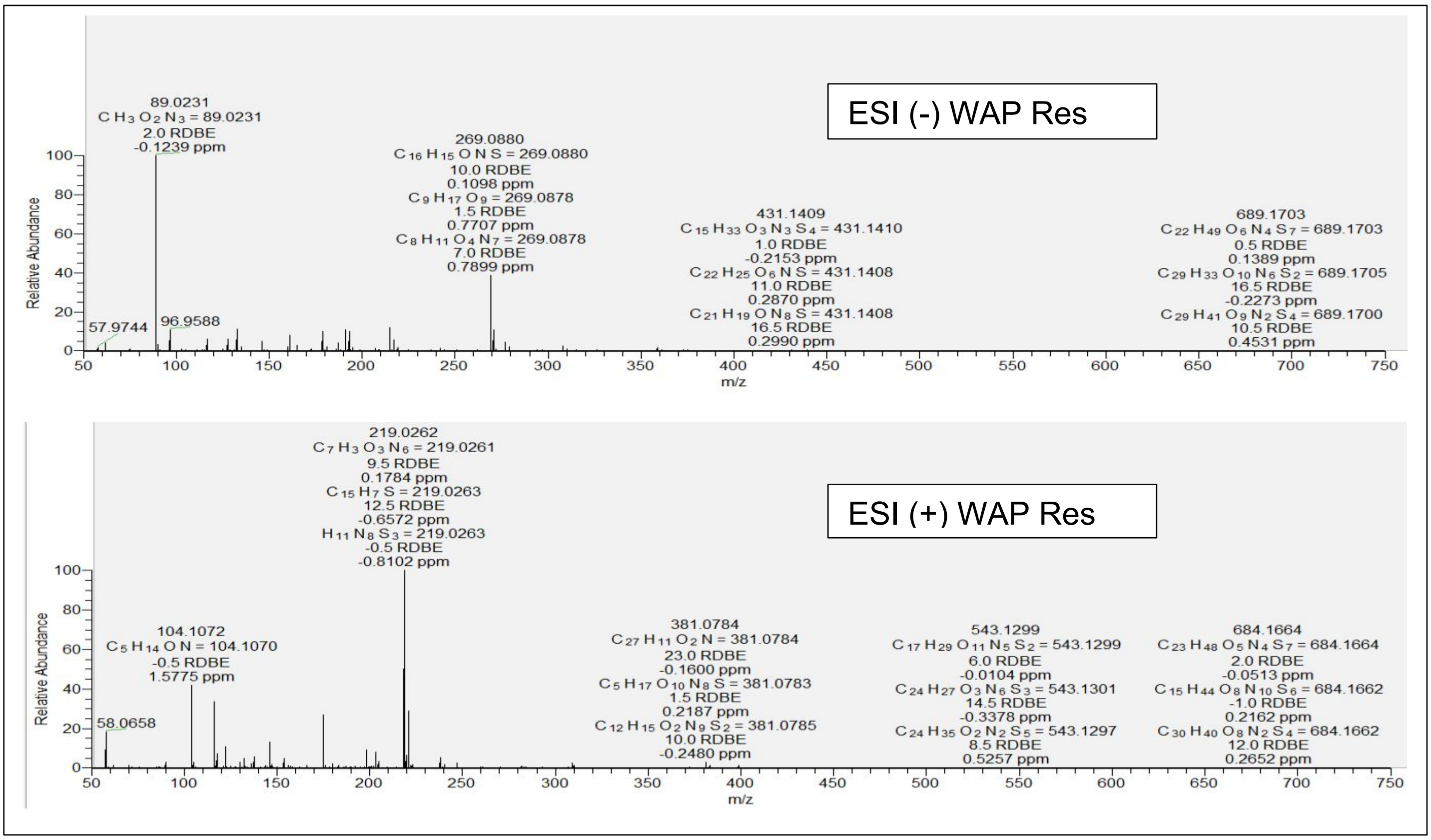

S10: Isotherms of Langmuir, Temkin, Flory-Huggins and El-Awady of the WEP

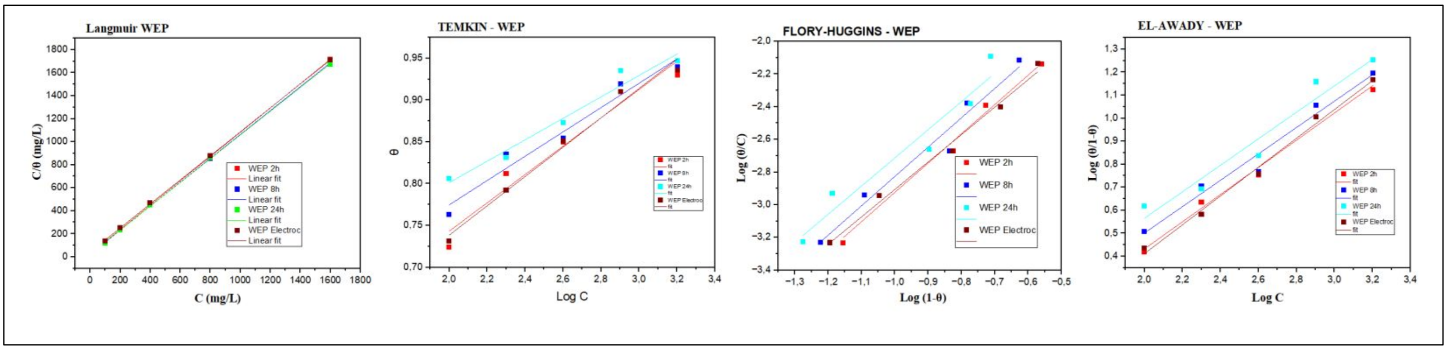

S11: Isotherms of Langmuir, Temkin, Flory-Huggins and El-Awady of the WAP

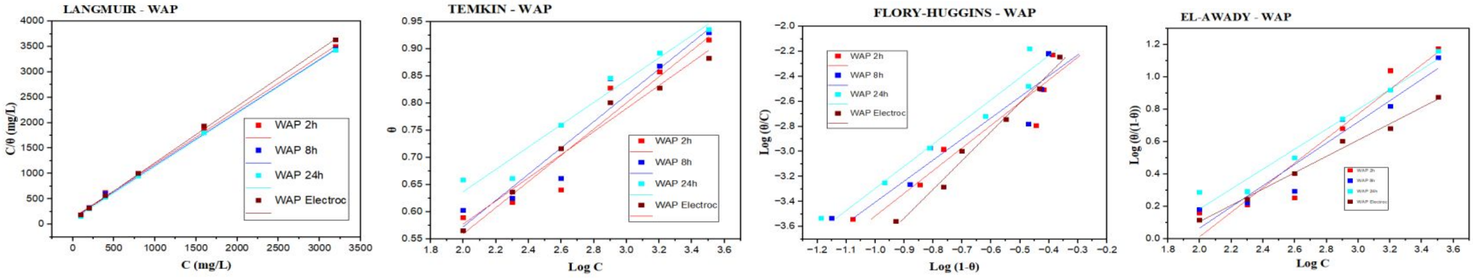

S12: XPS spectrum of WEP (Ethanolic extract of the *Armoracia rusticana*)

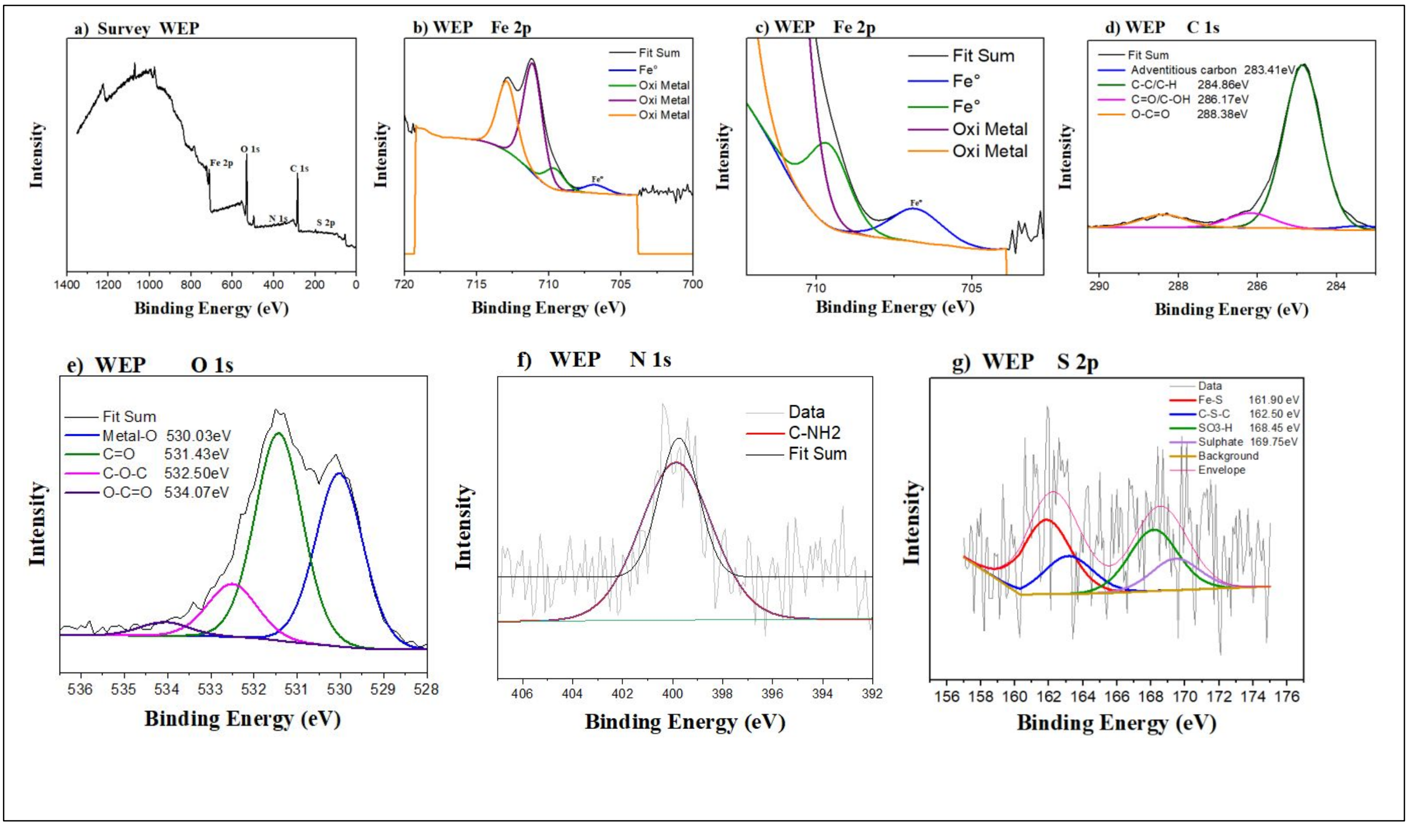

S13: XPS spectrum of WAP (Aqueous extract of the *Armoracia rusticana*)

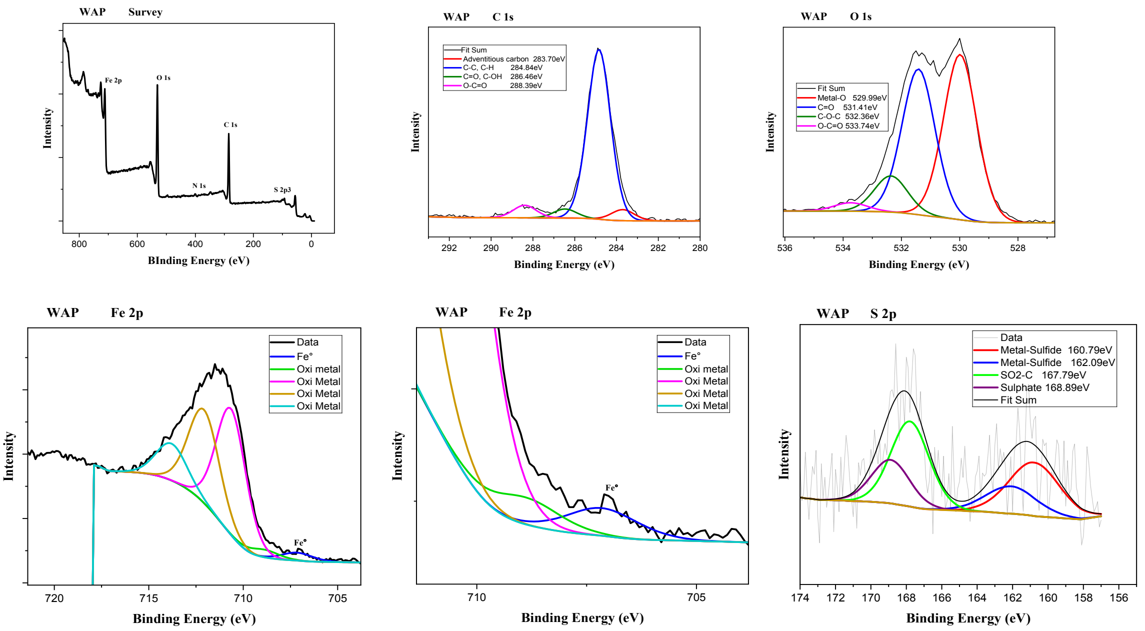

S14: Possible chemical structures of molecules adsorbed on the mild steel surface, identified by mass spectrometry MALDI-ICR-MS(±) and MALDI-TOF-MS(±)

Source: Author and pubchem.ncbi.nlm.nih.gov / (Elena Jimenez Negro a, 2022) / (CATHERINE H. BOTTING, 2002)

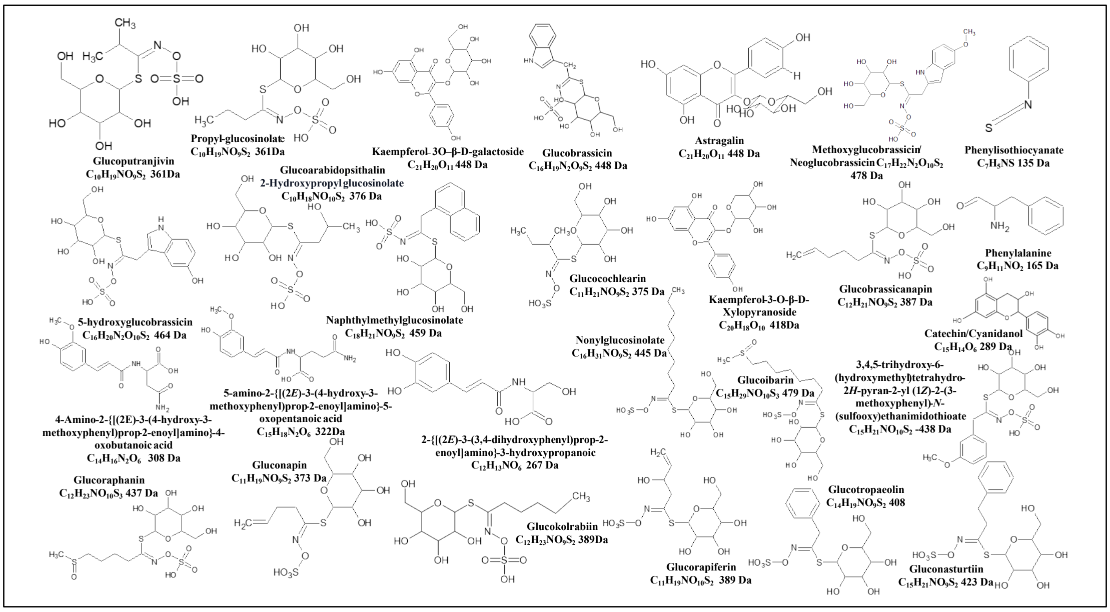

S15: Proposal for fragmentation (MS/MS (-)) of Glucobrassicin glucosinolate (422Da)

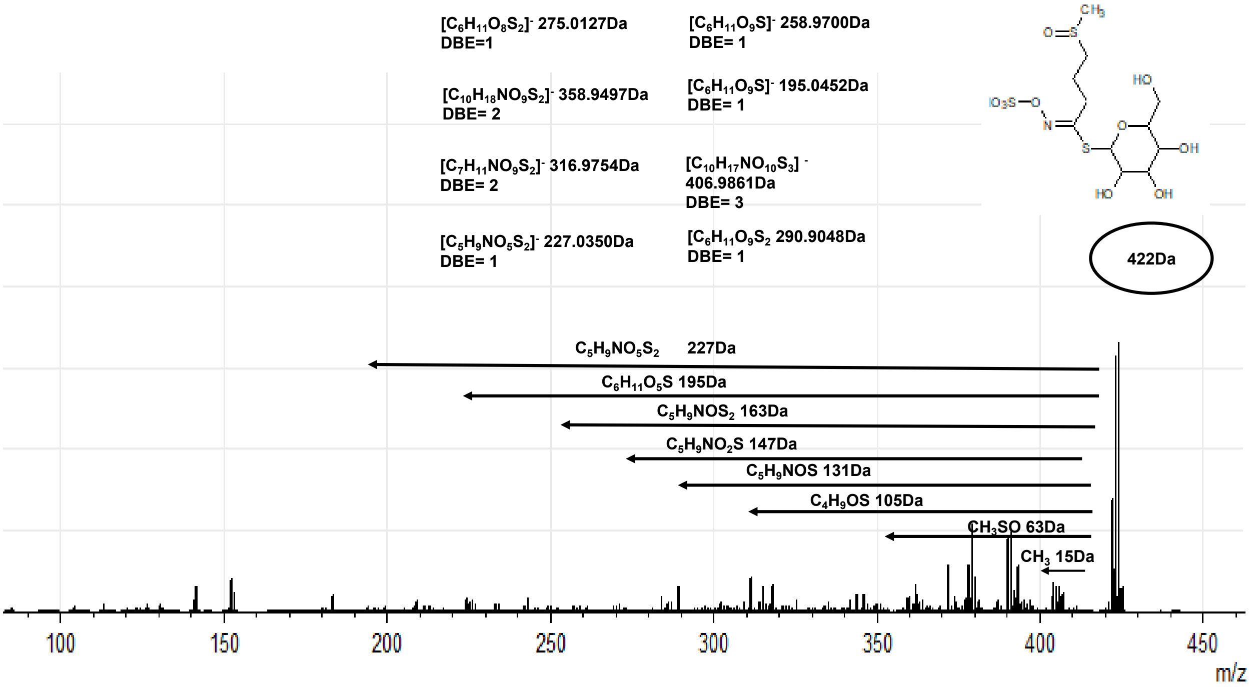

S16: Proposal for fragmentation (MS/MS (-)) of Sinigrin glucosinolate (358Da)

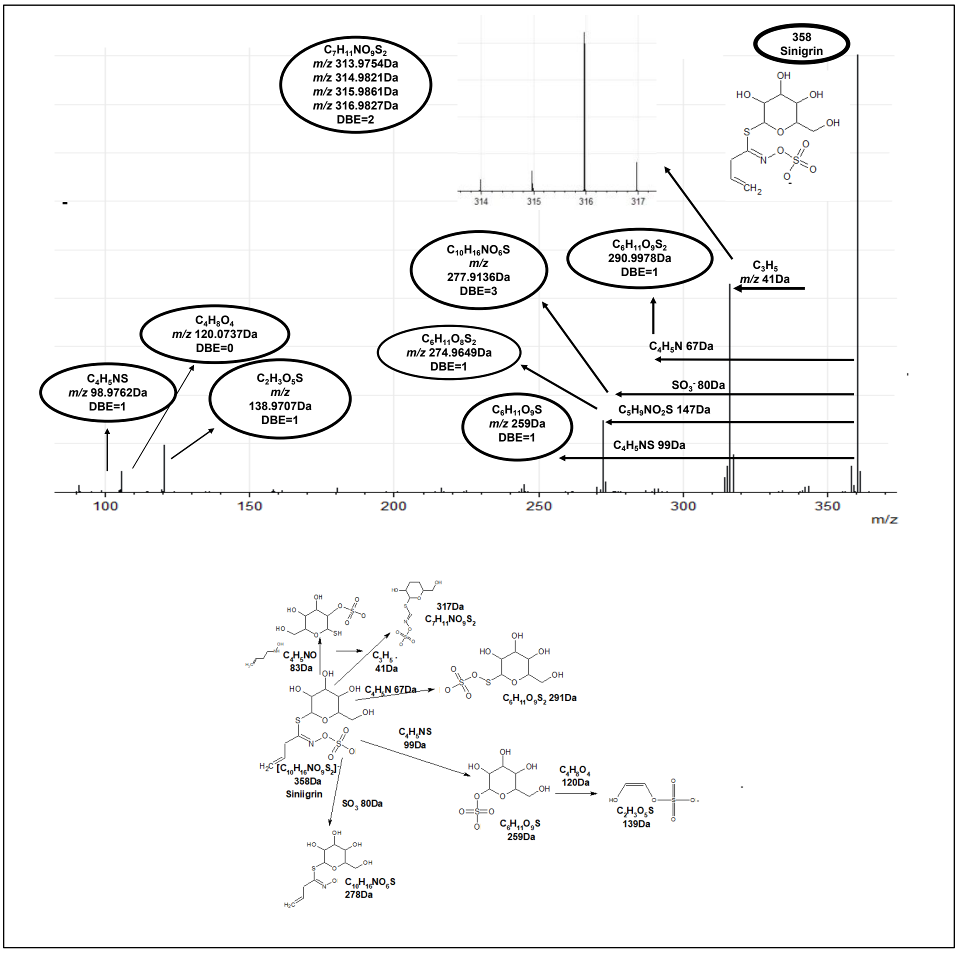

ST1: **ESI-QTOF/ESI-FTICR/MALDI-FTICR/MALDI-TOF** of Extract Ethanolic *Armoracia rusticana* - WEP – and fractions (Hexane, Ethyl acetate and Residual)

Propose strucutures identified in the WEP extract	Ion<br>$[M-H]^-$ / $[M+H]^+$	$m/z_{teórico}$<br>Da	m/z_{exp}<br>Da<br>ESI(±) / MALDI	Error<br>Da	DBE	Group
γ-aminobutyric acid	$[C_4H_{10}NO_2]^+$	104.0717	104.1071	0.0354	1	Organic acid
Benzaldehyde	$[C_7H_7O]^+$	107.0502	107.0360	-0.0142	5	Aldehyde
Benzyl alcohol	$[C_7H_9O]^+$	109.0659	109.0517	-0.0142	4	Organic acid
3-butenyl-isothiocyaate	$[C_5H_6NS]^-$	112.0226	112.0030	-0.0196	3	Isothiocyanate
Proline	$[C_5H_8NO_2]^-$<br>$[C_5H_{10}NO_2]^+$	114.0561<br>116.0706	114.0549<br>116.0707	-0.0012<br>-0.0001	2<br>2	Amino acid
Isobutyl-isothiocyanate	$[C_5H_8NS]^-$	114.0383	114.0410	0.0027	2	Isothiocyanate
Fumaric acid	$[C_4H_3O_4]^-$	115.0037	115.0389	0.0352	3	Organic acid
Succinic acid	$[C_4H_5O_4]^-$<br>$[C_4H_7O_4]^+$	117.0193<br>119.0339	117.0739<br>119.0896	0.0537<br>0.0557	2<br>2	Organic acid
Phenylacetaldehyde	$[C_8H_7O]^-$<br>$[C_8H_9O]^+$	119.0502<br>121.0648	119.0502<br>121.0647	0.0000<br>-0.0001	5<br>5	Aldehyde
Threonine	$[C_4H_9NO_3]^-$<br>$[C_4H_{11}NO_3]^+$	119.0588<br>121.0733	119.0491<br>121.0648	-0.0097<br>-0.0085	1<br>1	Amino acid
Cysteine	$[C_3H_8NO_2S]^+$	122.0270	122.0965	0.0695	1	Amino acid
Vinyl amyl ketone	$[C_8H_{13}O]^-$<br>$[C_8H_{15}O]^+$	125.0972<br>127.1117	125.0426<br>127.0754	-0.0546<br>-0.0363	1<br>1	Ketone
Naphthalene	$[C_{10}H_7]^-$<br>$[C_{10}H_9]^+$	127.0553<br>129.0699	127.0002<br>129.0521	-0.0551<br>-0,0178	7<br>7	Hidrocarbon
2-pentyl-isothiocyanate	$[C_6H_{10}NS]^-$	128.0539	128.0534	-0.0005	2	Isothiocyanate
Cyclopentyl isothiocyanate	$[C_6H_{10}NS]^-$<br>$[C_6H_{12}NS]^+$	128.0539<br>130.0685	128.1432<br>130.0861	0.0893<br>0.0199	3<br>3	Isothiocyanate
3-phenylpropionitrile	$[C_9H_8N]^-$	130.0662	130.0863	-0.0061	5	Miscellaneous
Asparagine	$[C_4H_7N_2O_3]^-$	131.0462	131.0339	-0.0123	2	Amino acid

	$[C_4H_9N_2O_3]^+$	133.0608	133.0428	-0.0180	2	
Malic acid	$[C_4H_5O_5]^-$<br>$[C_4H_7O_5]^+$	133.0142<br>135.0288	133.0132<br>135.0649	-0.0010<br>0.0361	2<br>2	Organic acid
4-ethylbenzaldeyde	$[C_9H_9O]^-$	133.0659	133.0496	-0.0163	5	Aldehyde
Phenylisothiocyanate	$[C_7H_5NS]^+$	136.0215	136.0209	-0.0006	6	Isothiocyanate
Anisaldeyde	$[C_8H_7O_2]^-$	135.0452	135.0460	0.0008	5	Aldehyde
α-phellandrene	$[C_{10}H_{15}]^-$<br>$[C_{10}H_{17}]^+$	135.1179<br>137.1325	135.0441<br>137.1546	-0.0738<br>0.0221	3<br>3	Terpenoid
p-salicylic acid	$[C_7H_5O_3]^-$	137.0244	137.0234	-0.0010	5	Organic acid
2-pentylfuran	$[C_9H_{13}O]^-$	137.0972	137.0268	-0.0704	3	Furan
Lysine	$[C_6H_{13}N_2O_2]^-$<br>$[C_6H_{15}N_2O_2]^+$	145.0983<br>147.1128	145.0610<br>147.1205	-0.0373<br>0.0077	1<br>1	Amino acid
benzylisothiocyanate	$[C_8H_8NS]^+$	152.0528	152.0457	-0.0071	6	Isothiocyanate
Vanillin	$[C_8H_7O_3]^-$<br>$[C_8H_9O_3]^+$	151.0401<br>153.0546	151.0423<br>153.0519	0.0022<br>-0.0027	5<br>5	Aldehyde
Menthol	$[C_{10}H_{19}O]^-$	155.1441	155.1070	-0.0371	1	Terpenoid
Erucin	$[C_6H_{10}NS_2]^-$	160.0260	160.0607	0.0347	2	Isothiocyanate
Phenethyl isothiocyanate	$[C_9H_8NS]^-$	162.0383	162.0480	0.0097	6	Isothiocyanate
Coumarinic acid	$[C_9H_7O_3]^-$<br>$[C_9H_9O_3]^+$	163.0401<br>165.0546	163.0392<br>165.0605	-0.0009<br>0.0059	6<br>6	Phenolic acid
Phenylalanine	$[C_9H_{10}NO_2]^-$	164.0717	164.0709	-0.0008	5	Amino acid
2-sec-butyl-methoxy-pyrazine	$[C_9H_{13}N_2O]^-$	165.1033	165.0742	-0.0291	4	Miscellaneous
Gallic acid	$[C_7H_5O_5]^-$<br>$[C_7H_7O_5]^+$	169.0142<br>171.0288	169.0166<br>171.0624	0.0024<br>0.0336	5<br>5	Phenolic acid
Arginine	$[C_6H_{13}N_4O_2]^-$	173.1044	173.1139	0.0095	2	Amino acid
Ascorbic acid	$[C_6H_7O_6]^-$	175.0248	175.0241	-0.0007	3	Organic acid
Esculetin	$[C_9H_5O_4]^-$<br>$[C_9H_7O_4]^+$	177.0182<br>179.0339	177.0338<br>179.0761	0.0156<br>0.0422	7<br>7	Coumarin
Caffeic acid	$[C_9H_7O_4]^-$	179.0350	179.0554	0.0204	6	Phenolic acid
Tyrosine	$[C_9H_{10}NO_3]^-$	180.0666	180.0660	-0.0006	5	Amino acid
Citric acid	$[C_6H_7O_7]^-$	191.0197	191.0191	-0.0006	3	Organic acid
Scopoletin	$[C_{10}H_7O_4]^-$<br>$[C_{10}H_9O_4]^+$	191.0350<br>193.0495	191.0393<br>193.0555	0.0043<br>0.0060	7<br>7	Coumarin
Syringic acid	$[C_9H_9O_5]^-$	197.0455	197.0424	-0.0031	5	Phenolic acid
Junipene	$[C_{15}H_{23}]^-$	203.1805	203.1112	-0.0693	4	Terpenoid
7-methylsulfinyl-heptyl-isothiocyanate	$[C_9H_{18}NOS_2]^+$	220.0835	220.0824	-0.0011	3	Isothiocyanate
Sinapinic acid	$[C_{11}H_{11}O_5]^-$	223.0612	223.0457	-0.0155	6	Phenolic acid
Myristic acid	$[C_{14}H_{27}O_2]^-$<br>$[C_{14}H_{29}O_2]^+$	227.2017<br>229.2162	227.1947<br>229.1990	-0.007<br>-0.0172	1<br>1	Fatty acid
13-hexadecenoic acid	$[C_{16}H_{29}O_2]^-$	253.2173	253.2097	-0.0076	2	Fatty acid
β-hydroxypalmitic acid	$[C_{16}H_{31}O_3]^-$	271.2279	271.2282	0.0003	1	Fatty acid
Pinolenic acid	$[C_{18}H_{29}O_2]^-$	277.2173	277.2175	0.0002	4	Fatty acid
Linoleic acid	$[C_{18}H_{31}O_2]^-$<br>$[C_{18}H_{33}O_2]^+$	279.2330<br>281.2475	279.2332<br>281.1422	0.0002<br>-0.1053	3<br>3	Fatty acid
Kaempferol	$[C_{15}H_9O_6]^-$	285.0405	285.0514	0.0109	11	Flavonoid
9,10-dihydroxypalmitic acid	$[C_{16}H_{31}O_4]^-$	287.2228	287.2232	0.0004	1	Fatty acid
Catechin	$[C_{15}H_{13}O_6]^-$<br>$[C_{15}H_{15}O_6]^+$	289.0718<br>291.0863	289.0681<br>291.0540	-0.0037	9<br>9	Flavonoid
17-hydroxyoleic acid	$[C_{18}H_{33}O_3]^-$	297.2435	297.2438	0.0003	2	Fatty acid
Sphingosine	$[C_{18}H_{36}NO_2]^-$	298.2752	298.2471	-0.0281	1	Miscellaneous
14-hydroxystearic acid	$[C_{18}H_{35}O_3]^-$	299.2592	299.2595	0.0003	1	Fatty acid
Quercetin	$[C_{15}H_9O_7]^-$	301.0354	301.0714	0.0360	11	Flavonoid
10,11-dihydroxyestearic acid	$[C_{18}H_{35}O_4]^-$	315.2541	315.0726	-0.1811	1	Fatty acid
Glucogallin	$[C_{13}H_{15}O_{10}]^-$<br>$[C_{13}H_{17}O_{10}]^+$	331.0671<br>333.0816	331.2142<br>333.1802	0.1470<br>0.0986	6<br>6	Tannin
Glucocapparin	$[C_8H_{14}NO_9S_2]^-$	332.0115	332.0741	0.0626	2	Glucosinolate
Glucolepidiin	$[C_9H_{17}NO_9S_2]^-$	346.0272	346.1259	0.0987	2	Glucosinolate
Chlorogenic acid	$[C_{16}H_{17}O_9]^-$	353.0878	353.0616	-0.0262	8	Phenolic acid
Sinigrin	$[C_{10}H_{16}NO_9S_2]^-$<br>$[C_{10}H_{18}NO_9S_2]^+$	358.0272<br>360.0417	358.0172<br>360.1493	-0.0100<br>0.1076	3<br>3	Glucosinolate
Propylglucosinolate	$[C_{10}H_{18}NO_9S_2]^-$	360.0428	360.0454	0.0026	2	Glucosinolate
Glucoputranjivin	$[C_{10}H_{18}NO_9S_2]^-$	360.0428	360.0919	0.0491	2	Glucosinolate
{[(2*E*)-3-(3,4-dihydroxyphenyl)prop-2-enoyl]amino}(1*H*-indol-3-yl)acetic acid	$[C_{20}H_{18}N_2O_5]^+$	366.1210	366.1080	-0.0130	7	Miscellaneous
Gluconapin	$[C_{11}H_{18}NO_9S_2]^-$	372.0428	372.1053	0.0625	3	Glucosinolate
Glucocochlearin (secbutyl)	$[C_{11}H_{20}NO_9S_2]^-$<br>$[C_{11}H_{22}NO_9S_2]^+$	374.0585<br>376.0730	374.0138<br>376.1252	-0.0447<br>0.0522	2<br>2	Glucosinolate
Butylglucosinolate	$[C_{11}H_{20}NO_9S_2]^-$	374.0585	374.0805	0.0220	2	Glucosinolate
Glucoconringianin (isobutyl)	$[C_{11}H_{20}NO_9S_2]^-$	374.0585	374.0952	0.0367	2	Glucosinolate
Glucosysimbrin	$[C_{10}H_{18}NO_{10}S_2]^-$	376.0378	376.0309	-0.0069	2	Glucosinolate

	$[C_{10}H_{20}NO_{10}S_2]^+$	378.0523	378.0726	0.0203	2	
Glucobrassicanapin	$[C_{12}H_{20}NO_9S_2]^-$	386.0585	386.0560	-0.0025	3	Glucosinolate
Pentylglucosinolate/Glucokholrabiin Glucojiaputin	$[C_{12}H_{22}NO_9S_2]^-$	388.0741	388.0301	-0.440	2	Glucosinolate
Glucorapiferin / Progoitrin	$[C_{11}H_{18}NO_{10}S_2]^-$<br>$[C_{11}H_{19}NO_{10}S_2]^+$	388.0378<br>390.0523	388.0301<br>390.0376	-0.0077<br>-0.0304	3<br>3	Glucosinolate
Glucoconringiin	$[C_{11}H_{20}NO_{10}S_2]^-$<br>$[C_{11}H_{22}NO_{10}S_2]^+$	390.0534<br>392.0680	390.0013<br>392.0643	-0.0521<br>-0.0037	2<br>2	Glucosinolate
Glucoiberverin	$[C_{11}H_{19}NO_9S_3]^-$<br>$[C_{11}H_{21}NO_9S_3]^+$	405.0227<br>407.0373	405.0902<br>407.0872	0.0675<br>0.0499	2<br>2	Glucosinolate
Glucotropaeolin	$[C_{14}H_{18}NO_9S_2]^-$	408.0428	408.1004	0.0576	6	Glucosinolate
Heptylglucosinolate	$[C_{14}H_{26}NO_9S_2]^-$	416.1054	416.1541	0.0487	2	Glucosinolate
Glucoiberin	$[C_{11}H_{20}NO_{10}S_3]^-$<br>$[C_{11}H_{22}NO_{10}S_3]^+$	422.0255<br>424.0400	422.0698<br>424.1297	0.0443<br>0.0897	2<br>2	Glucosinolate
Glucoberteroin	$[C_{13}H_{24}NO_9S_3]^-$	434.0619	434.2396	0.1777	2	Glucosinolate
Glucoraphanin	$[C_{12}H_{22}NO_{10}S_3]^-$	436.0411	436.0681	0.0270	2	Glucosinolate
Glucocheirolin	$[C_{11}H_{20}NO_{11}S_3]^-$<br>$[C_{11}H_{22}NO_{11}S_3]^+$	438.0204<br>440.0349	438.0932<br>440.0533	0.0728<br>0.0184	2<br>2	Glucosinolate
Glucobarbarin/Epiglucobarbarin	$[C_{15}H_{20}NO_{10}S_2]^-$	438.0534	438.0608	0.0074	6	Glucosinolate
Nonylglucosinolate	$[C_{16}H_{30}NO_9S_2]^-$	444.1367	444.1376	0.0009	2	Glucosinolate
Glucobrassicin	$[C_{16}H_{19}N_2O_9S_2]^-$	447.0537	447.0705	0.0168	8	Glucosinolate
Epigallocatechin Gallate	$[C_{22}H_{17}O_{11}]^-$	457.0776	457.1062	0.0286	14	Flavonoid
7-carboxymethyl-glucosinolate	$[C_{15}H_{26}NO_{11}S_2]^-$	460.0953	460.0928	-0.0025	3	Glucosinolate
5-hydroxyglucobrassicin<br>4- hydroxyglucobrassicin	$[C_{16}H_{19}N_2O_{10}S_2]^-$	463.0487	463.0961	0.0474	8	Glucosinolate
4-methoxyglucobrassicin	$[C_{17}H_{21}N_2O_{10}S_2]^-$	477.0643	477.1320	0.0677	8	Glucosinolate
Glucoibarin	$[C_{15}H_{29}NO_{10}S_3]^+$	480.0929	480.0934	0.0005		
Rutin	$[C_{27}H_{31}O_{16}]^+$	611.1607	611.1816	0.0209	13	Flavonoid
**Propose strucutures identified in the hexane fraction WEP extract**	**Ion [M-H]$^-$ / [M+H]$^+$**	***m/z*$_{teórico}$ Da**	***m/z*$_{exp}$ Da**	**Error Da**	**DBE**	**Group**
Benzaldehyde	$[C_7H_5O]^-$<br>$[C_7H_7O]^+$<br>$[C_7H_7O]^+$	105.0346<br>107.0502	105.0181<br>107.0019	-0.0165<br>-0.0483	5<br>5	Aldehyde
Benzyl alcohol	$[C_7H_7O]^-$<br>$[C_7H_9O]^+$	107.0502<br>109.0659	107.0497<br>109.0762	-0.0005<br>0.0103	4<br>4	Organic acid
Fumaric acid	$[C_4H_3O_4]^-$	115.0037	115.0025	-0.0012	3	Organic acid
Succinic acid	$[C_4H_5O_4]^-$	117.0193	117.0182	-0.0011	2	Organic acid
Naphtalene	$[C_{10}H_7]^-$<br>$[C_{10}H_8]^+$	127.0553<br>129.0699	127.0390<br>129.0852	-0.0163<br>0.0153	7<br>7	Hidrocarbon
3-phenylpropionitrile	$[C_9H_8N]^-$	130.0662	130.0132	-0.0530	5	Miscellaneous
Anisaldeyde	$[C_8H_7O_2]^-$	135.0452	135.0440	-0.0012	5	Aldehyde
α-phellandrene	$[C_{10}H_{15}]^-$<br>$[C_{10}H_{17}]^+$	135.1179<br>137.1325	135.1174<br>137.0961	-0.0005<br>-0.0364	3<br>3	Terpenoiod
2-pentylfuran	$[C_9H_{13}O]^-$<br>$[C_9H_{15}O]^+$	137.0972<br>139.1117	137.0717<br>137.1229	-0.0255<br>0.0112	3<br>3	Furan
benzylisothiocyanate	$[C_8H_8NS]^+$	152.0528	152.0457	-0.0071	6	Isothiocyanate
Menthol	$[C_{10}H_{19}O]^-$	155.1441	155.1338	-0.0103	1	Terpenoid
Phenethyl isothiocyanate	$[C_9H_8NS]^-$	162.0383	162.0302	-0.0081	6	Isothiocyanate
Coumarinic acid	$[C_9H_7O_3]^-$<br>$[C_9H_9O_3]^+$	163.0401<br>165.0546	163.0393<br>165.0608	-0.0008<br>0.0062	6<br>6	Phenolic acid
2-sec-butyl-methoxy-pyrazine	$[C_9H_{13}N_2O]^-$<br>$[C_9H_{15}N_2O]^+$	165.1033<br>167.1179	165.0582<br>167.1180	-0.0451<br>0.0001	4<br>4	Miscellaneous
Caffeic acid	$[C_9H_7O_4]^-$<br>$[C_9H_9O_4]^+$	179.0350<br>181.0495	179.0764<br>181.0532	0.0414<br>0.0037	6<br>6	Phenolic acid
Scopoletin	$[C_{10}H_7O_4]^-$<br>$[C_{10}H_9O_4]^+$	191.0350<br>193.0495	191.0326<br>193.0372	-0.0024<br>-0.0123	7<br>7	Coumarin
Syringic acid	$[C_9H_9O_5]^-$<br>$[C_9H_{11}O_5]^+$	197.0455<br>199.0601	197.0483<br>199.0399	0.0028<br>-0.0202	5<br>5	Phenolic acid
Junipene	$[C_{15}H_{23}]^-$<br>$[C_{15}H_{24}]^-$	203.1805<br>205.1951	203.1283<br>205.1700	-0.0522<br>-0.0251	4<br>4	Terpenoid
7-methylsulfinyl-heptyl-isothiocyanate	$[C_9H_{18}NOS_2]^+$	220.0835	220.1180	0.0345	3	Isothiocyanate
Myristic acid	$[C_{14}H_{27}O_2]^-$<br>$[C_{14}H_{29}O_2]^+$	227.2017<br>229.2162	227.2013<br>229.2159	-0.0004<br>-0.0003	1<br>1	Fatty acid
13-hexadecenoic acid	$[C_{16}H_{29}O_2]^-$<br>$[C_{16}H_{31}O_2]^+$	253.2174<br>255.2319	253.2162<br>255.2317	0.0157<br>-0.0002	2<br>2	Fatty acid
β-hydroxypalmitic acid	$[C_{16}H_{31}O_3]^-$<br>$[C_{16}H_{33}O_3]^+$	271.2279<br>273.2424	271.2281<br>273.2405	0.0002<br>-0.0019	1<br>1	Fatty acid
Pinolenic acid	$[C_{18}H_{29}O_2]^-$<br>$[C_{18}H_{31}O_2]^+$	277.2173<br>279.2319	277.2174<br>279.2319	0.0001<br>0.0000	4<br>4	Fatty acid

Linoleic acid	$[C_{18}H_{31}O_2]^-$<br>$[C_{18}H_{33}O_2]^+$	279.2330<br>281.2475	279.2319<br>281.2475	0.00011<br>0.0000	3<br>3	Fatty acid
9,10-dihydroxypalmitic acid	$[C_{16}H_{31}O_4]^-$	287.2228	287.1866	-0.0362	1	Fatty acid
17-hydroxyoleic acid	$[C_{18}H_{33}O_3]^-$	297.2435	297.1532	-0.0903	2	Fatty acid
Sphingosine	$[C_{18}H_{36}NO_2]^-$	298.2752	298.2477	-0.0275	1	Miscellaneous
14-hydroxystearic acid	$[C_{18}H_{35}O_3]^-$	299.2592	299.1828	-0.0764	1	Fatty acid
10,11-dihydroxyestearic acid	$[C_{18}H_{35}O_4]^-$	315.2541	315.2066	-0.0475	1	Fatty acid
Chlorogenic acid	$[C_{16}H_{17}O_9]^-$	353.0878	353.0729	-0.0149	8	Phenolic acid
{[(2*E*)-3-(3,4-dihydroxyphenyl)prop-2-enoyl]amino}(1*H*-indol-3-yl)acetic acid	$[C_{20}H_{18}N_2O_5]^+$	366.1210	366.1310	0.0100	7	Miscellaneous
**Propose strucutures identified in the Acetate fraction WEP extract**	**Ion<br>[M-H]$^-$ / [M+H]$^+$**	***m/z*$_{teórico}$<br>Da**	***m/z*$_{exp}$<br>Da**	**Error<br>Da**	**DBE**	**Group**
Proline	$[C_5H_8NO_2]^-$<br>$[C_5H_{10}NO_2]^+$	114.0561<br>116.0706	114.0549<br>116.0707	-0.0012<br>-0.0001	2<br>2	Amino acid
Phenylacetaldehyde	$[C_8H_7O]^-$<br>$[C_8H_9O]^+$	119.0502<br>121.0659	119.0491<br>121.0659	-0.0011<br>0.0000	5<br>5	Aldehyde
Threonine	$[C_4H_9NO_3]^-$<br>$[C_4H_{11}NO_3]^+$	119.0588<br>121.0733	119.0581<br>121.0619	-0.0007<br>-0.0114	1<br>1	Amino acid
2-pentyl-isothiocyanate	$[C_6H_{10}NS]^-$<br>$[C_6H_{12}NS]^+$	128.0539<br>130.0685	128.0495<br>130.0651	-0.0044<br>-0.0034	2<br>2	Isothiocyanate
Cyclopentyl isothiocyanate	$[C_6H_{10}NS]^-$<br>$[C_6H_{12}NS]^+$	128.0539<br>130.0685	128.0461<br>130.0651	0.0078<br>-0.0034	3<br>3	Isothiocyanate
Phenylisothiocyanate	$[C_7H_5NS + H]^+$	136.0215	136.0209	-0.0006	6	Isothiocyanate
Erucin	$[C_6H_{10}NS_2]^-$<br>$[C_6H_{12}NS_2]^+$	160.0260<br>162.0406	160.0244<br>162.0454	-0.0036<br>0.0048	2<br>2	Isothiocyanate
Kaempferol	$[C_{15}H_9O_6]^-$	285.0405	285.0408	0.0003	11	Flavonoid
Glucogallin	$[C_{13}H_{15}O_{10}]^-$<br>$[C_{13}H_{17}O_{10}]^+$	331.0671<br>333.0816	331.0626<br>333.0683	-0.0045<br>-0.0133	6<br>6	Tannin
Glucocapparin	$[C_8H_{14}NO_9S_2]^-$	332.0115	332.0273	0.0158	2	Glucosinolate
Glucolepidiin	$[C_9H_{17}NO_9S_2]^-$	346.0272	346.0915	0.0643	2	Glucosinolate
Sinigrin	$[C_{10}H_{16}NO_9S_2]^-$<br>$[C_{10}H_{18}NO_9S_2]^+$	358.0272<br>360.0417	358.0275<br>360.0452	0.0003<br>0.0035	3<br>3	Glucosinolate
Propylglucosinolate	$[C_{10}H_{18}NO_9S_2]^-$	360.0428	360.0615	0.0187	2	Glucosinolate
Gluconapin	$[C_{11}H_{18}NO_9S_2]^-$	372.0428	372.0577	0.0149	3	Glucosinolate
Glucocochlearin (secbutyl)	$[C_{11}H_{20}NO_9S_2]^-$<br>$[C_{11}H_{22}NO_9S_2]^+$	374.0585<br>376.0730	374.0567<br>376.0554	-0.0018<br>-0.0031	2<br>2	Glucosinolate
Butylglucosinolate	$[C_{11}H_{20}NO_9S_2]^-$<br>$[C_{11}H_{22}NO_9S_2]^+$	374.0585<br>376.0730	374.0567<br>376.0554	0.0220<br>-0.0031	2<br>2	Glucosinolate
Glucoconringianin (isobutyl)	$[C_{11}H_{20}NO_9S_2]^-$<br>$[C_{11}H_{22}NO_9S_2]^+$	374.0585<br>376.0730	374.0567<br>376.0554	0.0367-<br>0.0031	2<br>2	Glucosinolate
Glucosysimbrin	$[C_{10}H_{18}NO_{10}S_2]^-$<br>$[C_{10}H_{20}NO_{10}S_2]^-$	376.0378<br>378.0523	376.0508<br>378.0726	0.0013<br>0.0203	2<br>2	Glucosinolate
Glucobrassicanapin	$[C_{12}H_{20}NO_9S_2]^-$	386.0585	386.0557	-0.0028	3	Glucosinolate
Glucorapiferin / Progoitrin	$[C_{11}H_{18}NO_{10}S_2]^-$<br>$[C_{11}H_{19}NO_{10}S_2]^+$	388.0378<br>390.0523	388.0561<br>390.0624	-0.0077<br>0.0101	3<br>3	Glucosinolate
Glucoconringiin	$[C_{11}H_{20}NO_{10}S_2]^-$<br>$[C_{11}H_{22}NO_{10}S_2]^+$	390.0534<br>392.0680	390.0368<br>392.0510	-0.0166<br>-0.0170	2<br>2	Glucosinolate
Glucoiberverin	$[C_{11}H_{19}NO_9S_3]^-$	405.0227	405.0491	0.0264	2	Glucosinolate
Heptylglucosinolate	$[C_{14}H_{26}NO_9S_2]^-$	416.1054	416.1037	-0.0017	2	Glucosinolate
Glucoiberin	$[C_{11}H_{20}NO_{10}S_3]^-$	422.0255	422.0442	0.0187	2	Glucosinolate
Glucoberteroin	$[C_{13}H_{24}NO_9S_3]^-$	434.0619	434.0592	-0.0027	2	Glucosinolate
Glucoraphanin	$[C_{12}H_{22}NO_{10}S_3]^-$	436.0411	436.0892	0.0481	2	Glucosinolate
Nonylglucosinolate	$[C_{16}H_{30}NO_9S_2]^-$<br>$[C_{16}H_{32}NO_9S_2]^+$	444.1367<br>446.1513	444.1353<br>446.1558	-0.0014<br>0.0045	2<br>2	Glucosinolate
Glucobrassicin	$[C_{16}H_{19}N_2O_9S_2]^-$<br>$[C_{16}H_{21}N_2O_9S_2]^+$	447.0537<br>449.0683	447.0782<br>449.1085	0.0245<br>0.0402	8<br>8	Glucosinolate
Epigallocatechin Gallate	$[C_{22}H_{17}O_{11}]^-$<br>$[C_{22}H_{19}O_{11}]^+$	457.0776<br>459.0922	457.1039<br>459.0996	0.0263<br>0.0074	14	Flavonoid
7-carboxymethyl-glucosinolate	$[C_{15}H_{26}NO_{11}S_2]^-$	460.0953	460.0428	-0.0525	3	Glucosinolate
5-hydroxyglucobrassicin<br>4-hydroxyglucobrassicin	$[C_{16}H_{19}N_2O_{10}S_2]^-$	463.0487	463.0701	0.0214	8	Glucosinolate
4-methoxyglucobrassicin	$[C_{17}H_{21}N_2O_{10}S_2]^-$	477.0643	477.0468	-0.0175	8	Glucosinolate
Rutin	$[C_{27}H_{31}O_{16}]^+$	611.1607	611.1741	0.0134	13	Flavonoid
**Propose strucutures identified in the Residual fraction WEP extract**	**Ion<br>[M-H]$^-$ / [M+H]$^+$**	***m/z*$_{teórico}$<br>Da**	***m/z*$_{exp}$<br>Da**	**Error<br>Da**	**DBE**	**Group**
Oxalic acid	$[C_2HO_4]^-$	88.9880	88.9867	-0.0013	2	Organic acid
2-ethylfuran	$[C_6H_7O]^-$<br>$[C_6H_9O]^+$	95.0502<br>97.0648	95.0558<br>97.0651	0.0044<br>0.0003	3<br>3	Furan
Allyl isothiocyanate	$[C_4H_4NS]^-$	98.0070	98.0176	0.0106	2	Isothiocyanate

	$[C_4H_5NS]^+$	100.0215	100.0284	0.0069	2	
Isovaleric acid	$[C_5H_9O_2]^-$<br>$[C_5H_{10}O_2]^+$	101.0608<br>103.0754	101.0596<br>103.0869	-0.0012<br>0.0115	1<br>1	Organic acid
γ-aminobutyric acid	$[C_4H_8NO_2]^-$<br>$[C_4H_{10}NO_2]^+$	102.0561<br>104.0706	102.0548<br>104.0709	-0.0013<br>0.0003	1<br>1	Organic acid
3-buthenyl isothiocyanate	$[C_5H_6NS]^-$<br>$[C_5H_7NS]^+$	112.0226<br>114.0372	112.0212<br>114.0318	-0.0014<br>-0.0054	2<br>2	Isothiocyanate
Isobutyl-isothiocyanate	$[C_5H_8NS]^-$	114.0383	114.0366	-0.0017	2	Isothiocyanate
Cysteine	$[C_3H_6NO_2S]^-$	120.0125	120.0340	0.0215	1	Amino acid
Vinyl amyl ketone	$[C_8H_{13}O]^-$<br>$[C_8H_{15}O]^+$	125.0972<br>127.1117	125.0961<br>127.0754	-0.0011<br>-0.0363	1<br>1	Ketone
Asparagine	$[C_4H_7N_2O_3]^-$<br>$[C_4H_9N_2O_3]^+$	131.0462<br>133.0608	131.0452<br>133.0609	-0.0010<br>0.0000	2<br>2	Amino acid
Malic acid	$[C_4H_5O_5]^-$<br>$[C_4H_7O_5]^+$	133.0142<br>135.0288	133.0132<br>135.0299	-0.0010<br>0.0011	2<br>2	Organic acid
p-salicylic acid	$[C_7H_5O_3]^-$<br>$[C_7H_7O_3]^+$	137.0244<br>139.0390	137.0234<br>139.0292	-0.0010<br>0.0098	5<br>5	Organic acid
Lysine	$[C_6H_{13}N_2O_2]^-$<br>$[C_6H_{15}N_2O_2]^+$	145.0983<br>147.1128	145.0860<br>147.1127	-0.0123<br>-0.0001	1<br>1	Amino acid
Vanillin	$[C_8H_7O_3]^-$<br>$[C_8H_9O_3]^+$	151.0401<br>153.0546	151.0309<br>153.0546	-0.0092<br>0.0000	5<br>5	Aldehyde
Phenylalanine	$[C_9H_{10}NO_2]^-$<br>$[C_9H_{12}NO_2]^+$	164.0717<br>166.0863	164.0709<br>166.0861	-0.0008<br>-0.0002	5<br>5	Amino acid
Gallic acid	$[C_7H_5O_5]^-$<br>$[C_7H_7O_5]^+$	169.0142<br>171.0288	169.0135<br>171.0624	-0.0007<br>0.0336	5<br>5	Phenolic acid
Arginine	$[C_6H_{13}N_4O_2]^-$<br>$[C_6H_{14}N_4O_2]^+$	173.1044<br>175.1190	173.1139<br>175.1189	0.0095<br>-0.0001	2<br>2	Amino acid
Ascorbic acid	$[C_6H_7O_6]^-$<br>$[C_6H_8O_6]^-$	175.0248<br>177.0394	175.0241<br>177.0428	-0.0007<br>0.0034	3<br>3	Organic acid
Esculetin	$[C_9H_5O_4]^-$<br>$[C_9H_7O_4]^+$	177.0182<br>179.0339	177.0185<br>179.0761	0.0003<br>0.0422	7<br>7	Coumarin
Tyrosine	$[C_9H_{10}NO_3]^-$	180.0666	180.0660	-0.0006	5	Amino acid
Citric acid	$[C_6H_7O_7]^-$	191.0197	191.0192	-0.0005	3	Organic acid
Syringic acid	$[C_9H_9O_5]^-$	197.0455	197.0450	-0.0005	5	Phenolic acid
Sinapinic acid	$[C_{11}H_{11}O_5]^-$<br>$[C_{11}H_{13}O_5]^+$	223.0612<br>225.0757	223.0609<br>225.0690	-0.0003<br>-0.0067	6<br>6	Phenolic acid
Catechin	$[C_{15}H_{13}O_6]^-$<br>$[C_{15}H_{15}O_6]^+$	289.0718<br>291.0863	289.0762<br>291.0832	0.0044<br>-0.0031	9<br>9	Flavonoid
Quercetin	$[C_{15}H_9O_7]^-$	301.0354	301.0353	-0.0001	11	Flavonoid

ST2: Partition of Ethanolic Extract *of Armoracia rusticana* - WEP and fractions (Hexane, Ethyl Acetate and residual)

Propose strucutures identified in the WEP extract	Groups	WEP Hexane	WEP Acetate	WEP Residual
**γ-aminobutyric acid 103Da**	Organic acid			X
**Benzaldehyde 106Da**	Aldehyde	X		
**Benzyl alcohol 108Da**	Organic acid	X		
**3-butenyl-isothiocyaate 113Da**	Isothiocyanate			X
**Proline 115Da**	Amino acid		X	
**Isobutyl-isothiocyanate 115Da**	Isothiocyanate			X
**Fumaric acid 116Da**	Organic acid	X		
**Succinic acid 118Da**	Organic acid	X		
**Phenylacetaldehyde 119Da**	Aldehyde		X	
**Threonine 120Da**	Amino acid			X
**Cysteine 121Da**	Amino acid			X
**Vinyl amyl ketone 126Da**	Ketone			X
**Naphthalene 128Da**	Hidrocarbon	X		
**2-pentyl-isothiocyanate 129Da**	Isothiocyanate		X	

**Cyclopentyl isothiocyanate 129Da**	Isothiocyanate		X	
**3-phenylpropionitrile 131Da**	Miscellaneous	X		
**Asparagine 132Da**	Amino acid			X
**Malic acid 134Da**	Organic acid			X
**4-ethylbenzaldeyde 134Da**	Aldehyde			X
**Phenylisothiocyanate 135Da**	Isothiocyanate		X	
**Anisaldeyde 136Da**	Aldehyde	X		
**α-phellandrene 136Da**	Terpenoid	X		
**p-salicylic acid 138Da**	Organic acid			X
**2-pentylfuran 138Da**	Furan			X
**Lysine 146Da**	Amino acid			X
**Benzylisothiocyanate 151Da**	Isothiocyanate	X		
**Vanillin 152Da**	Aldehyde			X
**Menthol 156Da**	Terpenoid	X		
**Erucin 161Da**	Isothiocyanate		X	
**Phenethyl isothiocyanate 163Da**	Isothiocyanate	X		
**Coumarinic acid 164Da**	Phenolic acid	X		
**Phenylalanine 165Da**	Amino acid			X
**2-sec-butyl-methoxy-pyrazine 166Da**	Miscellaneous	X		
**Gallic acid 170Da**	Phenolic acid			X
**Arginine 174Da**	Amino acid			X
**Ascorbic acid 176Da**	Organic acid			X
**Esculetin 178Da**	Coumarin			X
**Caffeic acid 180Da**	Phenolic acid	X		
**Tyrosine 181Da**	Amino acid			X
**Citric acid 192Da**	Organic acid			X
**Scopoletin 192Da**	Coumarin	X		
**Syringic acid 198Da**	Phenolic acid			X
**Junipene 204Da**	Terpenoid	X		
**7-methylsulfinyl-heptyl-isothiocyanate 219Da**	Isothiocyanate	X		
**Sinapinic acid 224Da**	Phenolic acid			X
**Myristic acid 228Da**	Fatty acid	X		
**13-hexadecenoic acid 254Da**	Fatty acid	X		
**β-hydroxypalmitic acid 272Da**	Fatty acid	X		
**Pinolenic acid 278Da**	Fatty acid	X		
**Linoleic acid 280Da**	Fatty acid	X		
**Kaempferol 286Da**	Flavonoid		X	
**9,10-dihydroxypalmitic acid 288Da**	Fatty acid	X		
**Catechin 290Da**	Flavonoid			X
**17-hydroxyoleic acid 298Da**	Fatty acid	X		
**Sphingosine 299Da**	Miscellaneous	X		
**14-hydroxystearic acid 300Da**	Fatty acid	X		
**Quercetin 302Da**	Flavonoid			X
**10,11-dihydroxyestearic acid 316Da**	Fatty acid	X		
**Glucogallin 332Da**	Tannin		X	
**Glucocapparin 333Da**	Glucosinolate		X	

**Glucolepidiin 347Da**	Glucosinolate		X	
**Chlorogenic acid 354Da**	Phenolic acid	X		
**Sinigrin 359Da**	Glucosinolate		X	
**Propylglucosinolate 361Da**	Glucosinolate		X	
**Glucoputranjivin 361Da**	Glucosinolate		X	
**{[(2*E*)-3-(3,4-dihydroxyphenyl)prop-2-enoyl]amino}(1*H*-indol-3-yl)acetic acid 365Da**	Miscellaneous	X		
**Gluconapin 373Da**	Glucosinolate		X	
**Glucocochlearin (secbutyl) 375Da**	Glucosinolate		X	
**Butylglucosinolate 375Da**	Glucosinolate		X	
**Glucoconringianin (isobutyl) 375Da**	Glucosinolate		X	
**Glucosysimbrin 377Da**	Glucosinolate		X	
**Glucobrassicanapin 387Da**	Glucosinolate		X	
**Pentylglucosinolate/Glucokholrabiin Glucojiaputin 389Da**	Glucosinolate		X	
**Glucorapiferin / Progoitrin 389Da**	Glucosinolate		X	
**Glucoconringiin 391Da**	Glucosinolate		X	
**Glucoiberverin 406Da**	Glucosinolate		X	
**Glucotropaeolin 409Da**	Glucosinolate		X	
**Heptylglucosinolate 417Da**	Glucosinolate		X	
**Glucoiberin 423Da**	Glucosinolate		X	
**Glucoberteroin 435Da**	Glucosinolate		X	
**Glucoraphanin 437Da**	Glucosinolate		X	
**Glucocheirolin 439Da**	Glucosinolate		X	
**Glucobarbarin/Epiglucobarbarin 439Da**	Glucosinolate		X	
**Nonylglucosinolate 445Da**	Glucosinolate		X	
**Glucobrassicin 448Da**	Glucosinolate		X	
**Epigallocatechin Gallate 458Da**	Flavonoid		X	
**7-carboxymethyl-glucosinolate 461Da**	Glucosinolate		X	
**5-hydroxyglucobrassicin 464Da** 4-hydroxyglucobrassicin	Glucosinolate		X	
**4-methoxyglucobrassicin 478Da**	Glucosinolate		X	
**Rutin 610Da**	Flavonoid		X	

ST3: ESI-QTOF/ESI-FTICR/MALDI-FTICR/MALDI-TOF of Aqueous Extract of *Armoracia rusticana* - WAP and fractions (Hexane, Ethyl Acetate and Residual)

**Propose strucutures identified in the Hexane fraction WAP extract**	**Ion $[M-H]^-$ / $[M+H]^+$**	***$m/z_{teórico}$* Da**	***m/z_{exp}* Da**	**Error Da**	**DBE**	**Group**
Pentanal	$[C_5H_9O]^-$<br>$[C_5H_{11}O]^+$	85.0659<br>87.0808	85.0646<br>87.0804	-0.0013<br>-0.0004	1<br>1	Aldehyde
Oxalic acid	$[C_2HO_4]^-$	88.9880	88.9867	0.0013	2	Organic acid
2-ethylfuran	$[C_6H_7O]^-$<br>$[C_6H_9O]^+$	95.0502<br>97.0651	95.0490<br>97.0648	0.0012<br>0.0003	3<br>3	Furan
3-hexenal	$[C_6H_9O]^-$<br>$[C_6H_{11}O]^+$	97.0659<br>99.0807	97.0646<br>99.0804	-0.0013<br>0.0004	1<br>1	Aldehyde
Allyl isothiocyanate	$[C_4H_4NS]^-$	98.0070	98.0057	-0.0013	3	Isothiocyanate
Hexanal	$[C_6H_{11}O]^-$<br>$[C_6H_{13}O]^+$	99.0815<br>101.0961	99.0803<br>101.0963	-0.0012<br>0.0001	1<br>1	Aldehyde
Isovaleric acid	$[C_5H_9O_2]^-$<br>$[C_5H_{11}O_2]^+$	101.0597<br>103.0754	101.0595<br>103.0756	-0.0002<br>0.0002	1<br>1	Organic acid
γ-aminobutyric acid	$[C_4H_8NO_2]^-$	102.0550	102.0631	0.0081	1	Organic acid

	$[C_4H_{10}NO_2]^+$	104.0706	104.0708	0.0002	1	
Benzaldehyde	$[C_7H_5O]^-$	105.0346	105.0375	0.0029	5	Aldehyde
Benzyl alcohol	$[C_7H_9O]^+$	109.0648	109.0646	0.0002	4	Organic acid
3-butenyl isothiocyanate	$[C_5H_8NS]^+$	114.0372	114.0372	0.0000	3	Isothiocyanate
Proline	$[C_5H_8NO_2]^-$<br>$[C_5H_{10}NO_2]^+$	114.0549<br>116.0706	114.0549<br>116.0707	0.0000<br>0.0001	2<br>2	Amino acid
Succinic acid	$[C_4H_5O_4]^-$	117.0182	117.0181	-0.0001	2	Organic acid
Threonine	$[C_4H_8NO_3]^-$	118.0499	118.0497	0.0002	2	Amino acid
Phenylacetaldehyde	$[C_8H_8O]^-$	119.0491	119.0491	0.0000	5	Aldehyde
Cysteine	$[C_3H_6NO_2S]^-$	120.0125	120.0106	-0.0019	1	Amino acid
Vinyl amyl ketone	$[C_8H_{13}O]^-$<br>$[C_8H_{15}O]^+$	125.0961<br>127.1117	125.0961<br>127.1117	0.0000<br>0.0000	2<br>2	Ketone
3-phenylpropionitrile	$[C_9H_8N]^-$	130.0662	130.0863	-0.0201	5	Miscellaneous
Asparagine	$[C_4H_7N_2O_3]^-$	131.0462	131.0452	-0.0010	2	Amino acid
Vanillin	$[C_8H_7O_3]^-$	151.0401	151.0391	-0.0010	5	Aldehyde
Coumarinic acid	$[C_9H_7O_3]^-$	163.0390	163.0392	0.0002	6	Phenolic acid
Phenylalanine	$[C_9H_{10}NO_2]^-$<br>$[C_9H_{12}NO_2]^+$	164.0706<br>166.0862	164.0708<br>166.0861	0.0002<br>-0.0001	4<br>4	Amino acid
2-sec-butyl<br>-methoxy-pyrazine	$[C_9H_{14}N_2O]^+$	167.1179	167.1178	-0.0001	4	Miscellaneous
Ascorbic acid	$[C_6H_7O_6]^-$	175.0248	175.0241	-0.0007	3	Organic acid
13-hexadecenoic acid	$[C_{16}H_{29}O_2]^-$	253.2173	253.2175	0.0002	2	Fatty acid
Pinolenic acid	$[C_{18}H_{29}O_2]^-$<br>$[C_{18}H_{31}O_2]^+$	277.2173<br>279.2319	277.2174<br>279.2319	0.0001<br>0.0000	4<br>4	Fatty acid
Linoleic acid	$[C_{18}H_{31}O_2]^-$<br>$[C_{18}H_{33}O_2]^+$	279.2330<br>281.2475	279.2332<br>281.2472	0.0002<br>-0.0003	3<br>3	Fatty acid
Oleic acid	$[C_{18}H_{33}O_2]^-$	281.2486	281.2489	0.0003	2	Fatty acid
17-hydroxyoleic acid	$[C_{18}H_{33}O_3]^-$<br>$[C_{18}H_{35}O_3]^+$	297.2435<br>299.2581	297.2414<br>299.2577	-0.0010<br>-0.0004	2<br>2	Fatty acid
14-hydroxystearic acid	$[C_{18}H_{35}O_3]^-$	299.2592	299.2594	0.0002	1	Fatty acid
10,11-dihydroxyestearic acid	$[C_{18}H_{35}O_4]^-$	315.2541	315.2540	-0.0001	1	Fatty acid
Sinigrin	$[C_{10}H_{16}NO_9S_2]^-$<br>$[C_{10}H_{18}NO_9S_2]^+$	358.0272<br>360.0417	358.0274<br>360.1493	0.0002<br>0.1076	3<br>3	Glucosinolate
Gluconapin	$[C_{11}H_{18}NO_9S_2]^-$	372.0428	372.0426	-0.0002	3	Glucosinolate
Glucocochlearin (secbutyl)	$[C_{11}H_{20}NO_9S_2]^-$	374.0585	374.0587	0.0002	2	Glucosinolate
Glucoiberin	$[C_{11}H_{20}NO_{10}S_3]^-$	422.0255	422.0698	0.0443	2	Glucosinolate
**Propose strucutures identified in the Acetate fraction WAP extract**	**Ion**<br>**[M-H]- / [M+H]+**	***m/z*teórico**<br>**Da**	***m/z*exp**<br>**Da**	**Error**<br>**Da**	**DBE**	**Group**
Proline	$[C_5H_8NO_2]^-$<br>$[C_5H_{10}NO_2]^+$	114.0549<br>116.0706	114.0549<br>116.0707	0.0000<br>0.0001	2<br>2	Amino acid
Fumaric acid	$[C_4H_3O_4]^-$	115.0037	115.0025	-0.0012	3	Organic acid
Succinic acid	$[C_4H_5O_4]^-$	117.0186	117.0190	0.0004	2	Organic acid
Threonine	$[C_4H_8NO_3]^-$<br>$[C_4H_{10}NO_3]^+$	118.0499<br>120.0655	118.0498<br>120.0656	-0.0001<br>0.0001	1<br>1	Amino acid
Phenylacetaldehyde	$[C_8H_7O]^-$<br>$[C_8H_9O]^+$	119.0491<br>121.0659	119.0491<br>121.0659	0.0000<br>0.0000	5<br>5	Aldehyde
Cysteine	$[C_3H_6NO_2S]^-$	120.0115	120.0114	-0.0001	1	Amino acid
Asparagine	$[C_4H_7N_2O_3]^-$<br>$[C_4H_9N_2O_3]^+$	131.0451<br>133.0608	131.0451<br>133.0607	0.0000<br>-0.0001	2<br>2	Amino acid
4-ethylbenzaldeyde	$[C_9H_{11}O]^+$	135.0804	133.0804	0.0000	5	Aldehyde
Lysine	$[C_6H_{15}N_2O_2]^+$	147.1128	147.1127	0.0001	1	Amino acid
Vanillin	$[C_8H_7O_3]^-$	151.0401	151.0391	-0.0010	5	Aldehyde
Phenethyl isothiocyanate	$[C_9H_8NS]^-$	162.0383	162.0384	0.0001	6	Isothiocyanate
Coumarinic acid	$[C_9H_7O_3]^-$<br>$[C_9H_9O_3]^+$	163.0390<br>165.0546	163.0393<br>165.0545	0.0003<br>-0.0001	6<br>6	Phenolic acid
Phenylalanine	$[C_9H_{10}NO_2]^-$<br>$[C_9H_{12}NO_2]^+$	164.0706<br>166.0862	164.0708<br>166.0861	0.0002<br>-0.0001	4<br>4	Amino acid
Gallic acid	$[C_7H_5O_5]^-$<br>$[C_7H_7O_5]^+$	169.0142<br>171.0288	169.0134<br>171.0624	-0.0008<br>0.0336	5<br>5	Phenolic acid
Arginine	$[C_6H_{13}N_4O_2]^-$<br>$[C_6H_{15}N_4O_2]^+$	173.1044<br>175.1190	173.1036<br>175.1187	-0.0008<br>-0.0003	2<br>2	Amino acid
Ascorbic acid	$[C_6H_7O_6]^-$	175.0248	175.0240	-0.0008	3	Organic acid
Caffeic acid	$[C_9H_7O_4]^-$	179.0350	179.0345	-0.0005	6	Phenolic acid
Tyrosine	$[C_9H_{10}NO_3]^-$<br>$[C_9H_{12}NO_3]^+$	180.0666<br>182.0812	180.0658<br>180.0810	-0.0008<br>-0.0002	5<br>5	Amino acid
Citric acid	$[C_6H_7O_7]^-$	191.0197	191.0190	-0.0007	3	Organic acid
Syringic acid	$[C_9H_9O_5]^-$	197.0455	197.0448	-0.0007	5	Phenolic acid
Junipene	$[C_{15}H_{23}]^-$	203.1805	203.1817	0.0012	4	Terpenoid
Sinapinic acid	$[C_{11}H_{11}O_5]^-$	223.0612	223.0608	-0.0004	6	Phenolic acid

β-hydroxypalmitic acid	$[C_{16}H_{31}O_3]^-$	271.2279	271.2278	-0.0001	1	Fatty acid
**Propose strucutures identified in the Residual fraction WAP extract**	**Ion [M-H]$^-$ / [M+H]$^+$**	***m/z*$_{teórico}$ Da**	***m/z*$_{exp}$ Da**	**Error Da**	**DBE**	**Group**
Proline	$[C_5H_{10}NO_2]^+$	116.0706	116.0706	0.0000	2	Amino acid
p-salicylic acid	$[C_7H_5O_3]^-$	137.0233	137.0234	0.0001	5	Organic acid
Sinigrin	$[C_{10}H_{16}NO_9S_2]^-$	358.0272	358.0275	0.0003	3	Glucosinolate

ST4: Partition of Aqueous Extract of *Armoracia rusticana* – WAP and fractions (Hexane, Ethyl Acetate and Residual)

Propose strucutures identified in the WAP extract	Group	WAP Hexane	WAP Acetate	WAP Residual
Pentanal 86Da	Aldehyde	X		
Oxalic acid 90Da	Organic acid	X		
2-ethylfuran 96Da	Furan	X		
3-hexenal 98Da	Aldehyde	X		
Allyl isothiocyanate 99Da	Isothiocyanate	X		
Hexanal 100Da	Aldehyde	X		
Isovaleric acid 102Da	Organic acid	X		
γ-aminobutyric acid 103Da	Organic acid	X		
Benzaldehyde 106Da	Aldehyde	X		
Benzyl alcohol 108Da	Organic acid	X		
3-butenyl isothiocyanate 113Da	Isothiocyanate	X		
Proline 115Da	Amino acid	X	X	X
Fumaric acid 116Da	Organic acid		X	
Succinic acid 118Da	Organic acid	X	X	
Threonine 119Da	Amino acid	X	X	
Phenylacetaldehyde 120Da	Aldehyde	X	X	
Cysteine 121Da	Amino acid	X	X	
Vinyl amyl ketone 126Da	Ketone	X		
3-phenylpropionitrile 131Da	Miscellaneous	X		
Asparagine 132Da	Amino acid	X	X	
Malic acid 134Da	Organic acid		X	
p-salicylic acid 138Da	Organic acid			X
Lysine 146Da	Amino acid		X	
Vanillin 152Da	Aldehyde	X	X	
Phenethyl isothiocyanate 163Da	Isothiocyanate		X	
Coumarinic acid 164Da	Phenolic acid	X	X	
Phenylalanine 165Da	Amino acid	X	X	
2-sec-butyl -methoxy-pyrazine 166Da	Miscellaneous	X		
Gallic acid 170Da	Phenolic acid		X	
Arginine 174Da	Amino acid		X	
Ascorbic acid 176Da	Organic acid	X	X	
Caffeic acid 180Da	Phenolic acid		X	
Tyrosine 181Da	Amino acid		X	
Scopoletin 192Da	Coumarin		X	
Syringic acid 198Da	Phenolic acid		X	
Junipene 204Da	Terpenoid		X	
Sinapinic acid 224Da	Phenolic acid		X	
13-hexadecenoic acid 254Da	Fatty acid	X		
β-hydroxypalmitic acid 272Da	Fatty acid		X	
Pinolenic acid 278Da	Fatty acid	X		
Linoleic acid 280Da	Fatty acid	X		
Oleic acid 282Da	Fatty acid	X		
17-hydroxyoleic acid 298Da	Fatty acid	X		
14-hydroxystearic acid 300Da	Fatty acid	X		
10,11-dihydroxyestearic acid 316Da	Fatty acid	X		
Sinigrin 359Da	Glucosynolate	X		X
Gluconapin 373Da	Glucosynolate	X		
Glucocochlearin (secbutyl) 375Da	Glucosynolate	X		
Glucoiberin 423Da	Glucosynolate	X		

ST5: Langmuir, Temkin, Flory-Huggins and El-Awady isotherms equations of WEP and WAP

	Amostra	Langmuir	Temkin	Flory-Huggins	El-Alwady
**Mass loss measurements 2 h**	**WEP**	y= 1.0521x + 38.6375 R^2 = 0.9999	y = 0.1694x + 0.4044 R^2 = 0.9561	y = 1.7913x - 1.1329 R^2 = 0.9856	y = 0.5908x - 0.7502 R^2 = 0.9877
	**WAP**	y= 1.0518x + 125.4500 R^2 = 0.9977	y = 0.236x + 0.1019 R^2 = 0.9851	y = 1.6096x -1.7998 R^2 = 0.9203	y = 0.6654x – 1.2981 R^2 = 0.9164
**Mass loss measurements 8 h**	**WEP**	y= 1.0437x + 35.2454 R^2 = 0.9998	y = 0.1455x + 0.4836 R^2 = 0.9627	y = 1.7948x - 1.0320 R^2 = 0.9659	y = 0.5730x - 0.6453 R^2 = 0.9721
	**WAP**	y= 1.0594x +128.7523 R^2= 0.9984	y = 0.2421x + 0.0884 R^2 = 0.9246	y = 1.5061x - 1.8438 R^2 = 0.8952	y = 0.6573x - 1.2492 R^2 = 0.9249
**Mass loss measurements 24 h**	**WEP**	y= 1.0372x + 30.5502 R^2 = 0.9998	y = 0.1282x + 0.5448 R^2 = 0.9658	y = 1.7235x - 0.9897 R^2 = 0.9405	y = 0.5758x - 0.5867 R^2 = 0.9496
	**WAP**	y= 1.0468x + 91.4101 R^2 = 0.9995	y = 0.2055x + 0.2262 R^2 = 0.9625	y= 1.6836x – 1.5898 R^2 = 0.9518	y = 0.6161x - 1.0479 R^2 = 0.9650
**Electrochemical Impedance**	**WEP**	y= 1.0436x + 42.5549 R^2 = 0.9999	y= 0.1754x + 0.3876 R^2 = 0.9836	y= 1.6790x - 1.2273 R^2 = 0.9893	y= 0.6265x - 0.8419 R^2 = 0.9925
**Electrochemical Impedance**	**WAP**	y= 1.1110x + 102.2595 R^2 = 0.9994	y = 0.21279x + 0.1519 R^2 = 0.9794	y= 2.2930x – 1.4620 R^2 = 0.9877	y= 0.5042x - 0.9023 R^2 = 0.9921

ST6: Proposed structures of molecules possibly adsorbed on the surfaces of unwashed and washed plates, as well as in the wash water, identified by MALDI-FT-ICR-MS (±), MALDI-TOF-MS (±), and MALDI-TOF-MS/MS (±)

Propose structures identified in the WEP-EtOAc	Ion [M-H]$^-$ / [M+H]$^+$	$m/z_{teórico}$ Da	m/z_{exp} Da	Error Da	DBE
Phenylisothiocyanate	$[C_7H_5NS + H]^+$	136.0215 Da	136.0354 Da	0.0139 Da	6
Phenylalanine	$[C_9H_{11}NO_2 + H]^+$	166.0862 Da	165.9996 Da	-0.0866 Da	5
Tryptophan	$[C_{11}H_{12}N_2O_2 - H]^-$	203.0826Da	203.0760Da	-0,0066 Da	7
4-hydroxybrassicin isothiocyanate	$[C_{10}H_8N_2O_2S - H]^-$	203.0284 Da	203.0351 Da	0.0067 Da	8
Sinapic acid	$[C_{11}H_{12}O_5 - H]^-$	223.0612 Da	223.0434 Da	-0.0178 Da	6
Spirobrassicin	$[C_{11}H_{10}N_2OS_2 - H]^-$	249.0162 Da	249.0112 Da	0.0050 Da	8
4-Amino-4-oxo-2-{[(2E)-3-phenylprop-2-enoyl]amino}butanoic acid	$[C_{13}H_{14}N_2O_4 + H]^+$<br>$[C_{13}H_{14}N_2O_4 - H]^-$	263.1026 Da<br>261.0881 Da	262.9508 Da<br>261.0406 Da	0.1518 Da<br>-0.0475 Da	8
2-{[(2E)-3-(3,4-dihydroxyphenyl)prop-2-enoyl]amino}-3-hydroxypropanoic acid	$[C_{12}H_{13}NO_6 + H]^+$<br>$[C_{12}H_{13}NO_6 - H]^-$	268.0816 Da<br>266.0670	268.0352 Da<br>266.0586Da	-0,0464 Da<br>-0.0084 Da	7
Kaempferol or Luteolin 2-(3,4-dihydroxyphenyl)-5,7-dihydroxychromen-4-one	$[C_{15}H_{10}O_6 - H]^-$	285.0404 Da	285.0406 Da	0.0002 Da	11
Catechin/Cyanidol	$[C_{15}H_{14}O_6 + H]^+$	290.0785 Da	290.0422 Da	-0,0363 Da	9
Ácido 5-amino-2-{[(2*E*)-3-(4-hidroxifenil)prop-2-enoyl]amino}-5-oxopentanóico	$[C_{14}H_{15}N_2O_5 - H]^-$	291.0986Da	291.0944 Da	-0.0042 Da	8
2-{[(2E)-3-(4-hydroxy-3-methoxyphenyl)prop-2-enoyl]amino}-3-(4-hydroxyphenyl)propanoic acid	$[C_{13}H_{14}N_2O_6 + H]^+$<br>$[C_{13}H_{14}N_2O_6 - H]^-$	295.0925 Da<br>293.0779 Da	295.0228 Da<br>293.0759 Da	-0,0697 Da<br>-0.0020 Da	8
4-Amino-2-{[(2E)-3-(4-hydroxy-3-methoxyphenyl)prop-2-enoyl]amino}-4-oxobutanoic acid or 5-Amino-2-{[(2E)-3-(3,4-dihydroxyphenyl)prop-2-enol]amino}-5-oxopentanoic acid	$[C_{14}H_{16}N_2O_6 + H]^+$<br>$[C_{14}H_{16}N_2O_6 - H]^-$	309.1081 Da<br>307.0936 Da	309.0307 Da<br>307.0962 Da	0.0774 Da<br>0.0026 Da	8
5-amino-2-{[(2*E*)-3-(4-hydroxy-3-methoxyphenyl)prop-2-enoyl]amino}-5-oxopentanoic acid	$[C_{15}H_{18}N_2O_6 - H]^-$	321.1092 Da	321.0987 Da	-0.0105 Da	8
3-(3,4-dihydroxyphenyl)-2-{[(2E)- 3 -(4-hydroxyphenyl) prop-2-enoyl]amino} propanoic acid	$[C_{18}H_{17}NO_6 + H]^+$	344.1129 Da	344.1491 Da	-0.0362 Da	11
Sinigrim	$[C_{10}H_{17}NO_9S_2 - H]^-$	358.0272 Da	358.0336 Da<br>ms/ms<br>313.9754 Da<br>314.9821 Da<br>315.9861 Da<br>316.9827 Da<br>290.9978 Da<br>277.9136 Da<br>274.9649 Da<br>259.0119 Da	0.0064 Da	3

			138.9707 Da<br>120.0737 Da<br>98.9762 Da		
Glucoputranjivin or Propyl-glucosinolate	$[C_{10}H_{20}NO_9S_2 + H]^+$<br>$[C_{10}H_{19}NO_9S_2 - H]^-$	362.0574 Da<br>360.0428 Da	361.9739 Da<br>359.9951Da<br><br>361.9739 Da<br>ms/ms<br>317.9858Da<br>282.0061Da	-0.0835 Da<br>-0.0477 Da	2<br>2
Gluconapin	$[C_{11}H_{19}NO_9S_2 - H]^-$	372.0428 Da	372.0402 Da	-0.0026 Da	3
Glucocochlearin	$[C_{11}H_{21}NO_9S_2 + H]^+$<br>$[C_{11}H_{21}NO_9S_2 - H]^-$	376.0730 Da<br>374.0585 Da	375.9756 Da<br>374.0567 Da	-0.0975 Da<br>-0.0018 Da	2
7-Carboxy-methyl-glucosinolate	$[C_9H_{15}NO_{11}S_2 - H]^-$	376.0014 Da	376.0023 Da	-0.0009 Da	3
Glucokolrabiin or Glucojiaputin	$[C_{12}H_{23}NO_9S_2 - H]^-$	388.0741 Da	388.0714 Da	-0.0016 Da	2
Glucobrassicanapin	$[C_{12}H_{21}NO_9S_2 + H]^+$	388.0730 Da	388.0367 Da	-0.0364 Da	3
Glucorapiferin/Epi-Progoitrin	$[C_{11}H_{19}NO_{10}S_2 - H]^-$	388.0378 Da	388.0440 Da	0.0062 Da	3
Glucoconringiin or Glucocappariflexin (3,4,5-trihydroxy-6-(hydroxymethyl)tetrahydro-2*H*-pyran-2-yl (1*E*)-4-hydroxy-*N*-(sulfooxy)pentanimidothioate	$[C_{11}H_{21}NO_{10}S_2 - H]^-$	390.0534 Da	390.0368 Da	-0.0166 Da	2
Glucotropaeolin	$[C_{14}H_{19}NO_9S_2 - H]^-$	407.0428 Da	407.0375 Da	-0.0053 Da	6
Kaempferol-3-O-β-D-Xylopyranoside	$[C_{20}H_{18}O_{10} + H]^+$	419.0973 Da	419.0922 Da	-0.0051 Da	11
Glucoiberin	$[C_{11}H_{22}NO_{10}S_3 - H]^-$	422.0255 Da	422.0164 Da<br>ms/ms<br>406.9861 Da<br>358.9497 Da<br>316.9754 Da<br>290.9048 Da<br>274.9649 Da<br>258.9700 Da<br>227.0350 Da<br>195.0452 Da	-0.0091 Da	2
Gluconasturtiin (Phenethylglucosinolate)	$[C_{15}H_{21}NO_9S_2 - H]^-$	422.0585 Da	422.0567 Da	-0.0018 Da	3
Quercetin-3-θ-xyloside	$[C_{20}H_{18}O_{11} - H]^-$	433.0776 Da	433.0615 Da	-0.0161 Da	12
3,4,5-trihydroxy-6-(hydroxymethyl)tetrahydro-2*H*-pyran-2-yl (1*Z*)-2-(3-methoxyphenyl)-*N*-(sulfooxy)ethanimidothioate	$[C_{15}H_{21}NO_{10}S_2 - H]^-$	437.0456 Da	437.0517 Da	0.0061 Da	6
Glucoraphanin / 4-methylsulfinylbutylglucosinolate	$[C_{12}H_{24}NO_{10}S_3 + H]^+$<br>$[C_{12}H_{23}NO_{10}S_3 – H]^-$	438.0557 Da<br>436.0411 Da	438.0584 Da<br>436.0403 Da	0.0027 Da<br>-0.0008 Da	2
Epigallocatuhin Gallate	$[C_{12}H_{23}NO_{10}S_3 + H]^+$	438.0557 Da	438.5543 Da	0.0027 Da	2
3,4,5-trihydroxy-6-(hydroxymethyl)tetrahydro-2*H*-pyran-2-yl (1*Z*)-2-(3-methoxyphenyl)-*N*-(sulfooxy)ethanimidothioate	$[C_{15}H_{21}NO_{10}S_2 + H]^+$	439.0601 Da	439.0621 Da	0.0020 Da	6
**Glucobarbarin/Epiglucobarbarin** 3,4,5-trihydroxy-6-(hydroxymethyl)tetrahydro-2H-pyran-2-yl(1Z)-2-(3-methoxyphenyl)-N-(sulfooxy)ethanimidothioate	$[C_{15}H_{21}NO_{10}S_2 + H]^+$	440.0680 Da	440.0741 Da	0.0061 Da	6
Nonylglucosinolate	$[C_{16}H_{31}NO_9S_2 + H]^+$<br>$[C_{16}H_{31}NO_9S_2 - H]^-$	446.1513 Da<br>444.1367 Da	446.1110 Da<br>444.1418 Da	-0.0403 Da<br>0.0051 Da	2
Glucobrassicin	$[C_{16}H_{20}N_2O_9S_2 + H]^+$<br>$[C_{16}H_{19}N_2O_9S_2 - H]^-$	449.0683 Da<br>447.0537 Da	449.0510 Da<br>447.0421 Da	-0.0173 Da<br>-0.0116 Da	8

Kaempferol-3-O-β-D-glucoside or Kaempferol-3-O-B-D-galactoside Trifolin or Astragalin	$[C_{21}H_{20}O_{11} - H]^-$	447.0933 Da	447.0845 Da	-0.0088 Da	12
Naphthylmethylglucosinolate	$[C_{18}H_{21}NO_9S_2 + H]^+$	460.0730 Da	459.9915 Da	-0.0815 Da	9
7-Carboxy-methyl-glucosinolate	$[C_{15}H_{27}NO_{11}S_2 – H]^-$	460.0953 Da	460.0617 Da	-0.0336 Da	3
5-hydroxyglucobrassicin/ 4-hydroxyglucobrassicin	$[C_{16}H_{20}N_2O_{10}S_2 + H]^+$	465.0632Da	465.0572 Da	-0.0509 Da	8
Myricetin-3-O-Rhamnoside or Quercetin-3-0-β-D-glucoside	$[C_{21}H_{20}O_{12} + H]^+$	465.1028 Da	465.1004 Da	-0.0024 Da	12
4-Methoxyglucobrassicin Neoglucobrassicin	$[C_{17}H_{22}N_2O_{10}S_2 + H]^+$	479.0789 Da	479.0712 Da	-0.0077 Da	8
Brassicin	$[C_{22}H_{22}O_{12} + H]^+$	479.1184 Da	479.1012 Da	-0.0172 Da	12
Glucoibarin	$[C_{15}H_{29}NO_{10}S_3 + H]^+$	480.1026 Da	480.0929 Da	-0.0097Da	2
